# Innate and adaptive immune cell interaction drives inflammasome activation and hepatocyte apoptosis in murine liver injury from immune checkpoint inhibitors

**DOI:** 10.1038/s41419-024-06535-7

**Published:** 2024-02-14

**Authors:** Layla Shojaie, Jacob M. Bogdanov, Helia Alavifard, Mahmoud G. Mohamed, Aria Baktash, Myra Ali, Simeon Mahov, Sue Murray, Gary C. Kanel, Zhang-Xu Liu, Fumito Ito, Gino K. In, Akil Merchant, William Stohl, Lily Dara

**Affiliations:** 1 Division of Gastrointestinal and Liver Diseases, Department of Medicine, Keck School of Medicine of the University of Southern California, 2011 Zonal Avenue HMR 101, Los Angeles, CA 90033 USA; 2 Research Center for Liver Disease, Keck School of Medicine of the University of Southern California, 2011 Zonal Avenue HMR 101, Los Angeles, CA 90033 USA; 3 Division of Hematology and Cellular Therapy, Department of Medicine, Cedars-Sinai Medical Center, 127 S. San Vicente Boulevard Pavilion A8700, Los Angeles, CA 90048 USA; 4 Ionis Pharmaceuticals, Inc, 2855 Gazelle Ct, Carlsbad, CA 92010 USA; 5 Department of Pathology, Keck School of Medicine of the University of Southern California, 2011 Zonal Avenue HMR 211, Los Angeles, CA 90033 USA; 6 Translational Research Laboratory (TRLab), Alfred E. Mann School of Pharmacy and Pharmaceutical Sciences of the University of Southern California, 1985 Zonal Avenue, Los Angeles, CA 90033 USA; 7 Department of Surgery, Norris Comprehensive Cancer Center, University of Southern California Keck School of Medicine, 1441 Eastlake Avenue, Los Angeles, CA 90033 USA; 8 Division of Oncology, Department of Medicine, Norris Comprehensive Cancer Center, University of Southern California Keck School of Medicine, 1441 Eastlake Avenue, Los Angeles, CA 90033 USA; 9 Division of Rheumatology, Department of Medicine, Keck School of Medicine of the University of Southern California, 2011 Zonal Avenue HMR 711, Los Angeles, CA 90033 USA

**Keywords:** Pathogenesis, Molecular biology

## Abstract

Immune checkpoints (CTLA4 & PD-1) are inhibitory pathways that block aberrant immune activity and maintain self-tolerance. Tumors co-opt these checkpoints to avoid immune destruction. Immune checkpoint inhibitors (ICIs) activate immune cells and restore their tumoricidal potential, making them highly efficacious cancer therapies. However, immunotolerant organs such as the liver depend on these tolerogenic mechanisms, and their disruption with ICI use can trigger the unintended side effect of hepatotoxicity termed immune-mediated liver injury from ICIs (ILICI). Learning how to uncouple ILICI from ICI anti-tumor activity is of paramount clinical importance. We developed a murine model to recapitulate human ILICI using CTLA4^+/-^ mice treated with either combined anti-CTLA4 + anti-PDL1 or IgG1 + IgG2. We tested two forms of antisense oligonucleotides to knockdown caspase-3 in a total liver (parenchymal and non-parenchymal cells) or in a hepatocyte-specific manner. We also employed imaging mass cytometry (IMC), a powerful multiplex modality for immunophenotyping and cell interaction analysis in our model. ICI-treated mice had significant evidence of liver injury. We detected cleaved caspase-3 (cC3), indicating apoptosis was occurring, as well as Nod-like receptor protein 3 (NLRP3) inflammasome activation, but no necroptosis. Total liver knockdown of caspase-3 worsened liver injury, and induced further inflammasome activation, and Gasdermin-D-mediated pyroptosis. Hepatocyte-specific knockdown of caspase-3 reduced liver injury and NLRP3 inflammasome activation. IMC-generated single-cell data for 77,692 cells was used to identify 22 unique phenotypic clusters. Spatial analysis revealed that cC3+ hepatocytes had significantly closer interactions with macrophages, Kupffer cells, and NLRP3hi myeloid cells than other cell types. We also observed zones of three-way interaction between cC3+ hepatocytes, CD8 + T-cells, and macrophages. Our work is the first to identify hepatocyte apoptosis and NLRP3 inflammasome activation as drivers of ILICI. Furthermore, we report that the interplay between adaptive and innate immune cells is critical to hepatocyte apoptosis and ILICI.

## Introduction

Cytotoxic T-lymphocyte associated protein-4 (CTLA-4) and programmed cell death protein-1 (PD-1) are checkpoint receptors expressed on the surface of cytotoxic T cells (CTL) that interact with their respective ligands CD80/CD86 and PD-1 ligand (PD-L1). These inhibitory pathways maintain self-tolerance by attenuating T-lymphocyte activation and maintaining immune responses within a desired physiologic range. Malignant tumors co-opt these immune suppressive mechanisms to avoid immune destruction [1]. The liver has tolerogenic mechanisms in place via both the adaptive and innate immune systems to prevent aberrant immune activation [2–5]. PD-L1 expression by hepatocytes and innate liver non-parenchymal cells (NPC) helps induce T-cell apoptosis and exhaustion and can be upregulated in the setting of inflammation to dampen immune activation [2–4]. CTLA-4 reduces inflammatory cytokine production in CTLs causing exhaustion and simultaneously promotes the formation of anti-inflammatory CD4^+^FOXP3^+^ regulatory T-cells (Tregs) [2–6]. When these pathways are disrupted, the intricately balanced immunotolerant environment in the liver gives way to unchecked and dysregulated immune activation [3–9]. This results in unintended side effects such as hepatotoxicity or immune-mediated liver injury from ICI (ILICI) [10–13]. Patients with high-grade ILICI require discontinuation of immunotherapy and initiation of high-dose steroids which have deleterious effects on tumor growth. Therefore, developing targeted therapies to treat ILICI and resume immunotherapy is of paramount clinical importance [13–15].

ICIs increase the clonal expansion of self-recognizing CTLs, increase Th1 and Th17 numbers at the expense of Tregs, and promote the production of pro-inflammatory cytokines [4–6, 9]. Myeloid cells are exquisitely sensitive to a pro-inflammatory milieu and become significant producers of inflammatory cytokines and serve as a nidus for immune cell activation [2–6, 9, 16]. Accordingly, several groups have recently suggested that both adaptive and innate immune cells participate in liver injury secondary to ICI use [4, 7–9]. Gudd et al. showed that in patients who developed ILICI increased pro-inflammatory myeloid cell recruitment to the liver contributed to hepatotoxicity [8]. Despite these important associations between the innate and adaptive systems, there is yet no understanding of the underlying mechanisms that lead to hepatocyte death in this context.

We set out to investigate the events leading to ILICI focusing on the modes of programmed cell death, with the goal of identifying which cells are dying, how, and why. We developed a novel murine ILICI model and observed activation of apoptosis and the NLRP3 inflammasome, along with infiltration of adaptive and innate immune cells. Apoptosis is classically the cell death pathway induced by CTLs and is detected by the cleavage of caspase-3 [17]. Pyroptosis is an inflammatory mode of cell death executed by the pore-forming cleaved N-terminal of Gasdermin-D (GSDMD-N), leading to the release of cleaved IL-1β [17]. Pyroptosis is activated by the innate immune system and plays a significant role in maintaining and propagating the inflammatory milieu [17–19]. Activation of the Nod-like receptor protein 3 (NLRP3) inflammasome, a multi-protein complex that acts as an inflammatory sensor, is associated with liver inflammation upstream of GSDMD cleavage and pyroptosis [18–23]. Specifically, NLRP3 is activated in hepatocytes and myeloid cells and drives the homing of additional immune cells to inflamed tissue by promoting cytokine secretion [18–20, 22]. Here we show that hepatocyte apoptosis is a feature of ILICI and blocking apoptosis in the entire liver population (parenchymal and non-parenchymal cells) results in increased inflammasome activation and fuels pyroptosis, worsening liver injury. Conversely, hepatocyte-targeted apoptosis inhibition protects from ILICI. Using imaging mass cytometry (IMC) for multiplex immunophenotyping in mouse liver we show that NLRP3^hi^ myeloid cells drive hepatocyte death.

## Results

### Murine model of immune-mediated liver injury from checkpoint inhibitors (ILICI)

**Fig. 1:**
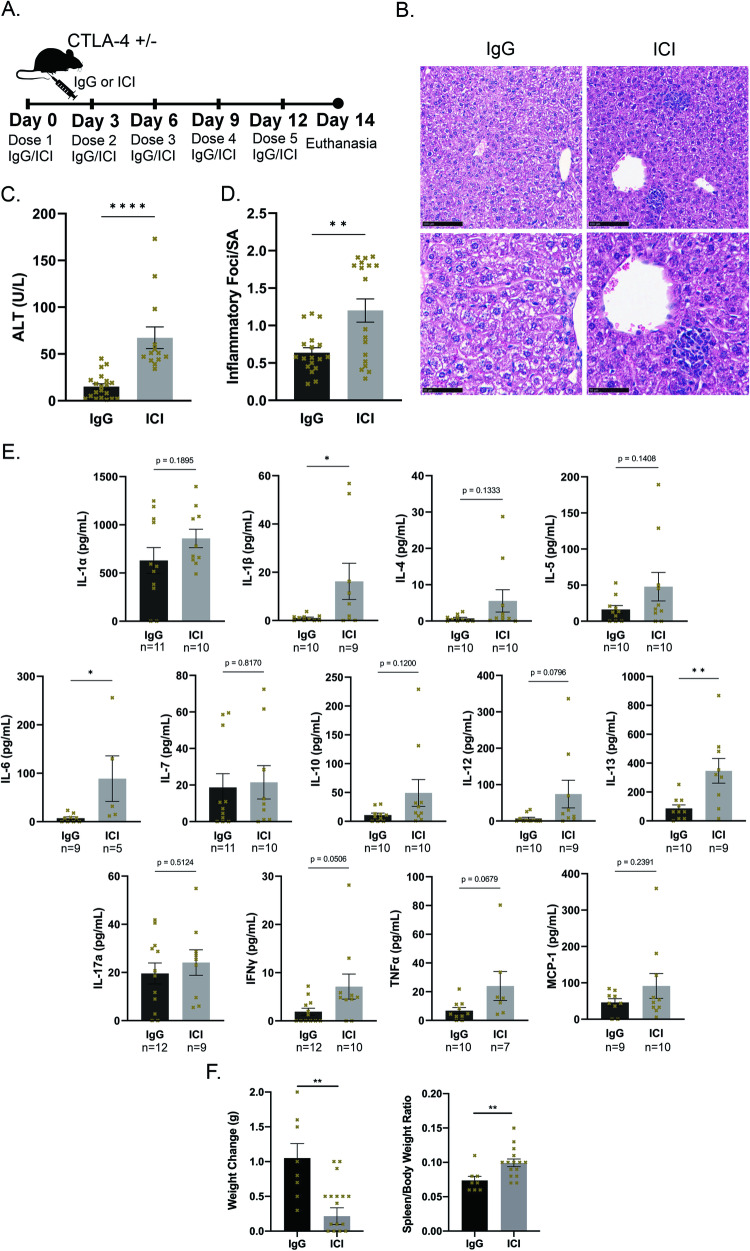
Immune checkpoint inhibitors (ICI) induce liver disease in a murine injury model. **A** Murine injury model of immune-mediated liver injury from ICIs (ILICI). 8–12 week-old CTLA4^+/-^ littermates received anti-CTLA4 plus anti-PD-L1 or isotype control (IgG1 and IgG2) via intraperitoneal injection as shown **B** Representative H&E images Top: 20x, Bottom: 40x **C** Alanine aminotransferase (ALT) *n* = 13 for ICI, 20 for IgG. **D** Quantification of inflammatory foci over surface area in liver sections *n* = 18/group. **E** Cytokines. **F** Quantification of (left) body weight change and (right) spleen/body weight ratio between day 0 and day 14 *n* = 8 IgG and 16 ICI. Data are mean ± SEM. *P*-value calculated using unpaired *t*-test. *P*-value * (<0.05), ** (<0.01), *** (<0.001), **** (<0.0001).

We developed a new animal model of ILICI using CTLA-4 haploinsufficient (CTLA-4^+/-^) mice that received either combined ICI treatment with anti-CTLA4 + anti-PD-L1 antibodies, or isotype IgG over a 14-day period (Fig. 1A). ICI-treated mice displayed histological and biochemical evidence of liver injury and had significantly more necro-inflammatory liver foci and higher serum alanine aminotransferases (ALT) compared to IgG-treated mice (Fig. 1B–D). While CTLA^+/+^ mice also exhibited liver injury in response to ICIs, the haploinsufficient animals produced a more consistent and robust injury phenotype (Sup. Fig. 1A–C). On histology, the necro-inflammatory foci with hepatocyte dropout most commonly occurred in zone 2 of the liver and consisted not only of lymphocytes but also macrophages (Sup. Fig. 1D, E). Immunohistochemistry (IHC) (Sup. Fig. 1F) demonstrated these inflammatory foci to stain positive for CD4, CD8, and F4/80.

To further characterize the inflammatory environment, we conducted a Luminex cytokine assay to measure cytokines (Fig. 1E). Serum levels of all thirteen cytokines tested were higher in ICI-treated mice than in IgG-treated mice, with the differences in levels of Interleukin (IL) -1β, IL-6, and IL-13 achieving statistical significance. ICI-treated mice also gained significantly less weight than IgG-treated mice and had significantly increased spleen/body weight ratios at the time of euthanasia (Fig. 1F). These findings support the generation of ICI-induced liver injury and inflammation in our mouse model.

### Apoptosis is activated in the livers of ICI-treated mice

**Fig. 2:**
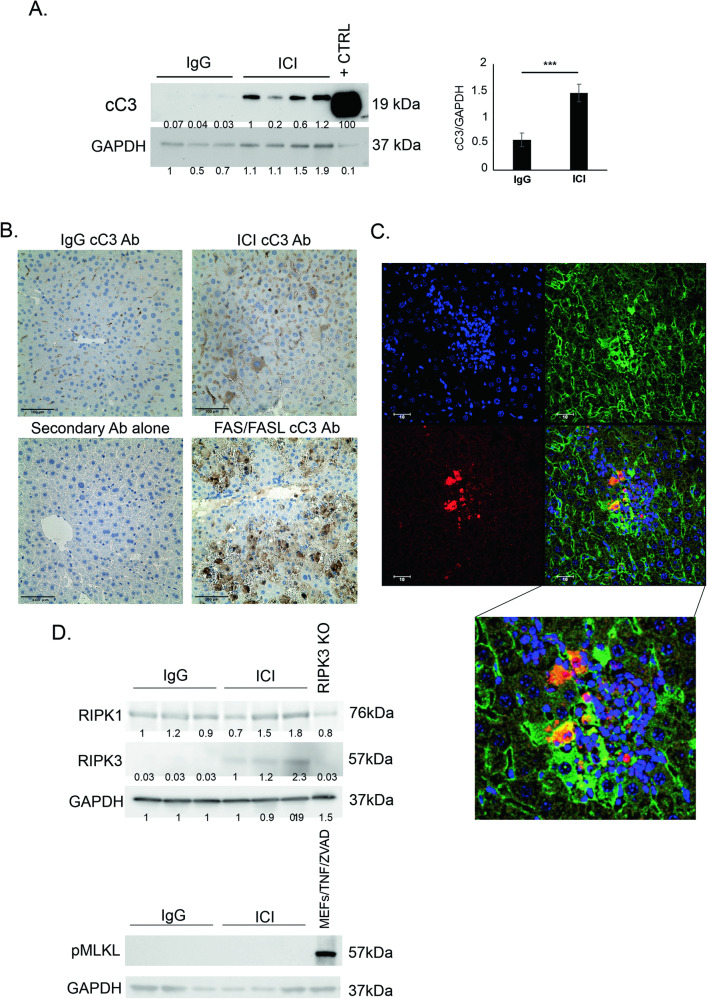
Apoptosis is activated in the livers of ICI-treated mice. **A** (left) Representative western blot from whole liver lysates of treated CTLA4^+/-^ mice showing detection of cleaved Caspase-3 in the ICI-treated group, and loading control. Densitometry (right) *n* = 3 IgG and 4 ICI. **B** Immunohistochemistry for cleaved Caspase-3. 20x magnification. **C** Representative immunofluorescence for cleaved Caspase-3 and HNF4-α signals 40x magnification. **D** Western blot from whole liver lysates of treated CTLA4^+/-^ mice for RIPK1, RIPK3, pMLKL, and GAPDH (RIPK3 KO was used as internal WB control negative to show antibody specificity). Data are mean ± SEM. *P*-value calculated using unpaired *t*-test. *P*-value * (<0.05).

In determining the mode of cell death, we first tested the most common form of liver cell death, apoptosis. Immunoblotting of whole liver extracts showed increased cC3, the activated executioner caspase of apoptosis, in ICI-treated mice (Fig. 2A). IHC revealed that hepatocytes of ICI-treated mice stained positively for cC3 (Fig. 2B). Immunofluorescence with Hepatocyte nuclear factor 4- α (HNF4-α)/cC3/ 4’,6-diamidino-2-phenylindole (DAPI) co-staining demonstrated dying hepatocytes with cytoplasmic cC3 signal next to inflammatory foci (Fig. 2C). As seen here, these larger cC3+ cells had the morphologic appearance and size of hepatocytes and were in proximity to shrinking apoptotic bodies reflecting other dying cells further along the death process.

Next, we tested for activation of necroptosis, an alternative cell death pathway involving the activation of receptor-interacting protein kinases 1 and 3 (RIPK1 and RIPK3) leading to phospho-activation of the effector protein mixed lineage kinase domain-like (pMLKL), which disrupts the plasma membrane [17]. We did not detect any pMLKL, indicating the absence of appreciable necroptosis (Fig. 2D). We did see an increase in RIPK3 expression in whole liver lysates after ICI treatment which likely corresponds to increased infiltrating immune cells. We and others have previously shown that leukocytes express high levels of RIPK3 while hepatocytes do not. Therefore, the increased RIPK3 in total liver lysates reflects the immune infiltrate [24, 25]. We conducted further experiments with a modified ILICI model using mixed lineage kinase domain-like (MLKL) knockout (MLKL^-/-^) mice (Sup. Fig. 2A). These mice underwent the same ICI treatment and had more foci of inflammation on liver sections than their IgG control counterparts. Genetic elimination of MLKL therefore was not protective in our injury model, suggesting necroptosis is not a key player in ILICI (Sup. Fig. 2B, C). In summation, we detected hepatocyte apoptosis in the livers of ICI-treated mice, but no necroptosis.

### The NLRP3 inflammasome is activated in the livers of ICI-treated mice

**Fig. 3:**
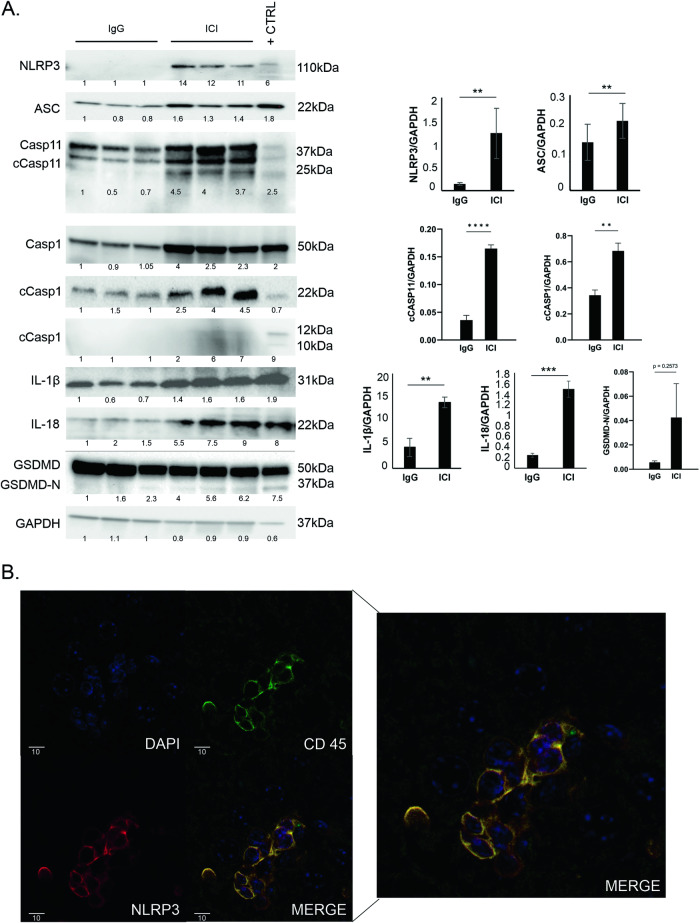
Activation of the NLRP3 inflammasome in the livers of ICI-treated mice. **A** (left) Representative western blot from whole liver lysates of treated CTLA4^+/-^ mice for indicated proteins and loading control (GAPDH). (right) Densitometry. **B** Immunofluorescence for NLRP3 and CD45 on CTLA4^+/-^ liver treated with ICI, and overlay (bottom-right, enlarged in cutout). 40x magnification. Data are mean ± SEM *n* = 6/group. *P*-values were calculated using an unpaired *t*-test. *P*-value ** (<0.01), *** (<0.001), **** (<0.0001).

Activation of the NLRP3 inflammasome contributes to various inflammatory liver diseases [18–23]. An NLRP3 gain-of-function mutation alone in macrophages or neutrophils is sufficient in inducing autoinflammation in the liver [19]. We next tested whether NLRP3 inflammasome activation or pyroptotic cell death was occurring in our ILICI model. Interestingly, NLRP3, Apoptosis speck-like protein (ASC), IL-18 and IL-1β levels were all significantly increased in the ICI-treated group. Furthermore, significantly more cleavage of both caspase-1 and caspase-11 was detected in the ICI-treated group indicating the activation of both the canonical and non-canonical pathways, an observation that has been described in other contexts [26–28]. Gasdermin-D cleavage (GSDMD-N) was not consistent and not sufficiently increased (Fig. 3A), indicating that NLRP3-mediated inflammation is occurring without pyroptosis. Immunofluorescence co-staining revealed several hepatocytes with cytoplasmic NLRP3 signal (not shown), as well as abundant NLRP3 signal in hepatic non-parenchymal immune cells (CD45+) (Fig. 3B). Taken together, these observations indicate that the NLRP3 inflammasome is activated in the livers of ICI-treated mice.

### Total liver Caspase-3 knockdown via antisense oligonucleotide (ASO) worsens liver injury in ICI-treated mice

**Fig. 4:**
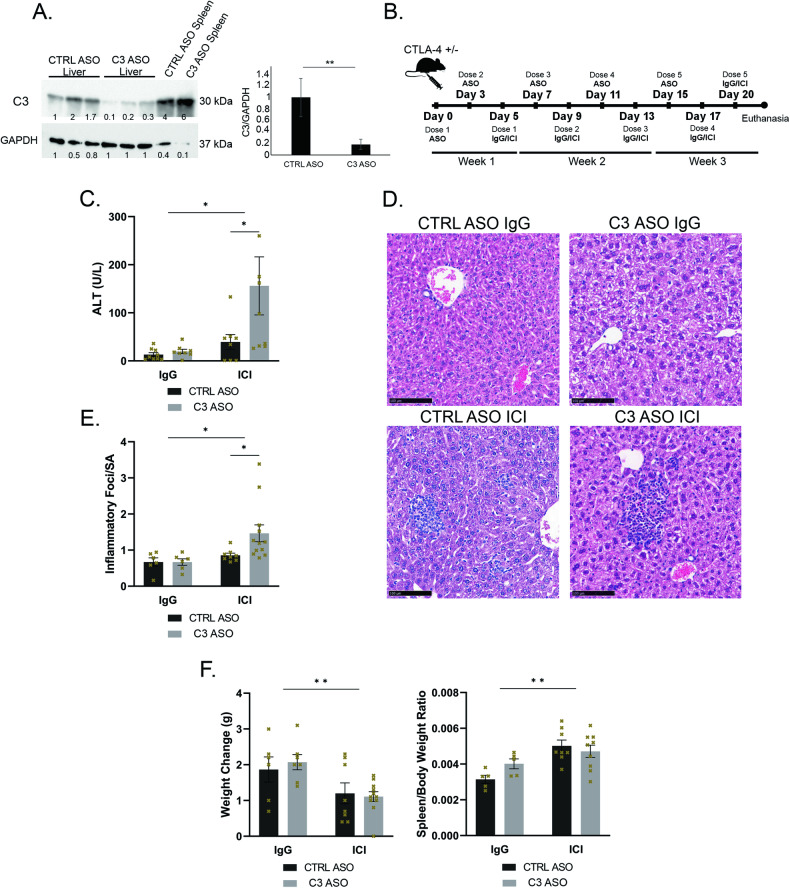
Total liver Caspase-3 knockdown via unconjugated antisense oligonucleotide (ASO) worsens liver injury in ICI-treated mice. **A** (left) Representative western blot of whole liver and spleen lysates of CTLA4^+/-^ demonstrating knockdown of Caspase-3. (right) Densitometry *n* = 3/group. Data are mean ± SEM. *P*-value calculated using unpaired *t*-test. **B** Murine injury model of ILICI modified to include ASO treatment. CTLA4^+/-^ littermates were treated with either CTRL-ASO or C3-ASO for two doses prior to receiving isotype control IgG or ICI treatment alternating with ASO. **C** ALT. Data are mean ± SEM. n = 8 CTRL IgG and 9 for all other groups. *P*-values were calculated using a two-way analysis of variance (ANOVA) followed by Sidak’s test for multiple comparisons. **D** Representative H&E images 20x magnification. **E** Quantification of inflammatory foci over surface area in liver sections. Data are mean ± SEM. *n* = 6 IgGs and 9 ICIs. *P*-values were calculated using two-way ANOVA followed by Sidak’s test for multiple comparisons. **F** Quantification of (left) body weight change and (right) spleen/ body weight ratio between day 0 and day 21. *n* = 6 for IgG, 8 CTRL-ASO ICI, 11 C3-ASO ICI. Data are mean ± SEM. *P*-values were calculated using two-way ANOVA. *P*-value *(<0.05), **(<0.01).

To further probe the importance of apoptosis in ILICI, we used ASO to knockdown liver caspase-3 (C3-ASO). ASO binds to complementary mRNA transcripts of target genes, preventing translation, and resulting in protein knockdown. C3-ASO accumulates primarily in the liver [24, 29] and knocks down caspase-3 expression in the entire liver cell population (parenchymal and NPCs). We confirmed that the antisense knocked down caspase-3 in the liver but not the spleen, and by itself did not induce any liver injury (Fig. 4A and Sup. Fig. 3A). We modified our murine ILICI model to incorporate a C3-ASO or control-ASO (CTRL-ASO) treatment in addition to our standard treatments with either combined ICIs or IgG controls over a 21-day period (Fig. 4B). ICI-treated mice again displayed histological and biochemical evidence of immune-mediated liver injury. Furthermore, total liver knockdown of caspase-3 worsened the degree of injury on histology and ALT in ICI-treated mice (Fig. 4C–E) and did not affect weight changes seen with ICI (Fig. 4F). Therefore, complete knockdown of caspase-3 in liver parenchymal cells and NPCs did not ameliorate liver injury and in fact exacerbated it.

### Hepatocyte-specific knockdown of Caspase-3 using GalNac-conjugated ASO protects from ILICI in mice

**Fig. 5:**
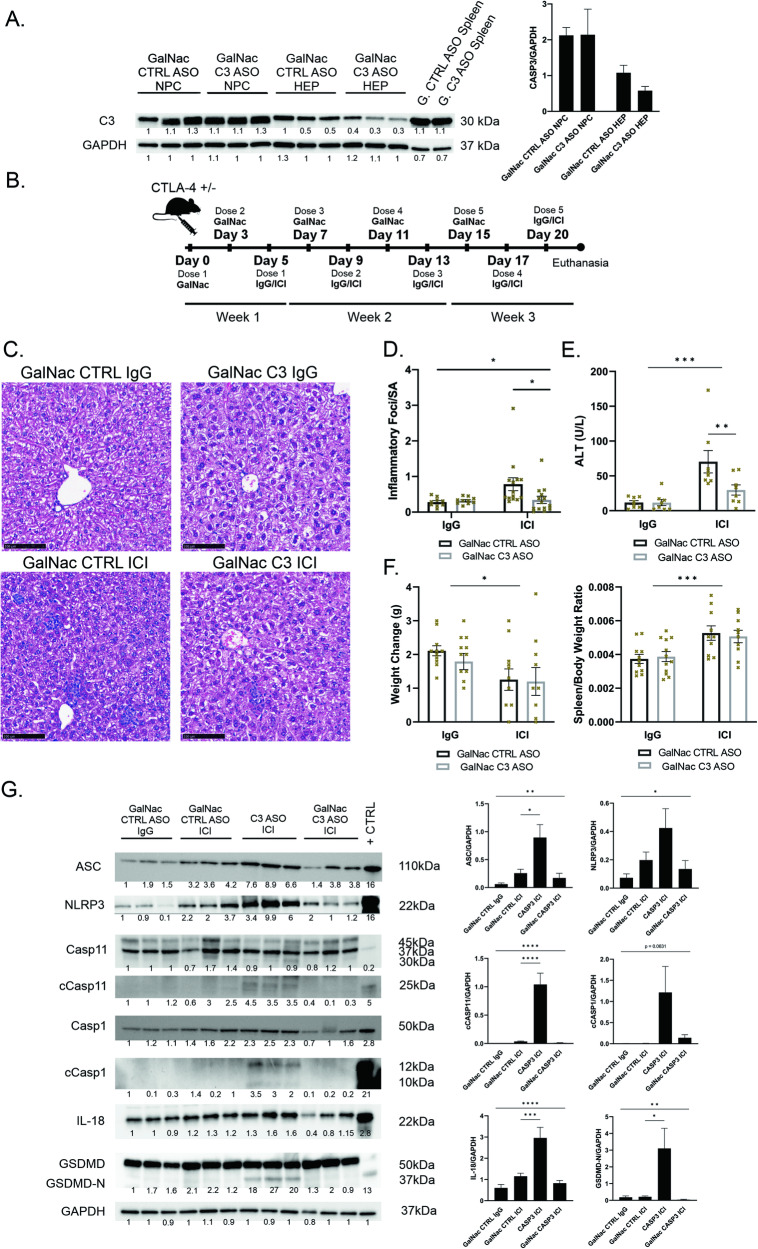
Hepatocyte-specific Caspase-3 knockdown using GalNac-ASO ameliorates liver injury in ICI-treated mice. **A** (left) Hepatocytes (HEP) and non-parenchymal cells (NPC) were isolated from livers of CTLA4^+/-^ mice treated with GalNac-control-ASO and GalNac-Caspase-3-ASO, homogenized, and immunoblotting was performed for total Caspase-3 and GAPDH (loading control) to demonstrate hepatocyte-specific knockdown. (right) Densitometry. Data are mean ± SEM. *n* = 3/group. **B** Murine injury model of ILICI modified to include GalNac-ASO treatment. CTLA4^+/-^ littermates were treated with either GalNac-CTRL-ASO or GalNac-C3-ASO for two doses prior to receiving isotype control IgG or ICI treatment alternating with GalNac-ASO. **C** Representative H&E images 20x magnification. **D** Quantification of inflammatory foci over the surface area in liver sections *n* = 9 IgG, 14 GN-CTRL + ICI, 12 GN-C3 + ICI. **E** ALT *n* = 8/group. **F** Quantification of (left) body weight change and (right) spleen/body weight ratio between day 0 and day 21. Data are mean ± SEM n = 11 GN-IgGs, 10 GN-CTRL + ICI, 9 GN-C3 + ICI. **G** (left) Representative western blot of whole liver lysates of treated CTLA4^+/-^ mice for indicated proteins. (right) Densitometry *n* = 6/group. *P*-values were calculated using one-way or two-way ANOVA followed by Sidak’s or Tukey’s test for multiple comparisons when appropriate. *P*-value *(<0.05), **(<0.01), ***(<0.001), ****(<0.0001).

We next sought to knockdown hepatocyte caspase-3 using GalNac-conjugated ASO [29]. The asialoglycoprotein receptor (ASGPR), is a C-type lectin that is abundantly expressed on hepatocytes. GalNac-conjugated ASO enhances ASGPR binding and increases the total and fraction of administered ASO dose to hepatocytes. GalNac-ASOs are also more potent and can be delivered to hepatocytes at much lower doses [29]. To test for hepatocyte-specific caspase-3 knockdown, GalNAc-CTRL-ASO and GalNac-C3-ASO-treated livers underwent collagenase perfusion to isolate the NPC and hepatocyte compartments. Lysates of liver NPCs and hepatocytes from ASO-treated mice demonstrated hepatocyte but not NPC caspase-3 knockdown (Fig. 5A). Having confirmed hepatocyte-specific knockdown, we incorporated GalNac-C3-ASO or GalNac-CTRL-ASO into our model (Fig. 5B).

In contrast to total liver knockdown of caspase-3, hepatocyte-specific caspase-3 knockdown lessened the severity of liver injury. Livers of GalNac-C3-ASO + ICI-treated mice had less inflammatory foci/Surface area (SA) when compared to livers of GalNac-CTRL-ASO + ICI-treated mice (Fig. 5C, D). Serum ALT concentrations were decreased in GalNac-C3-ASO + ICI-treated mice compared to the GalNac-CTRL-ASO + ICI group (Fig. 5E). As in the unconjugated ASO experiments, GalNac-ASO treatment by itself in the absence of ICIs caused no discernible liver damage and did not affect weight changes seen with ICI (Fig. 5F).

### Total liver Caspase-3 knockdown increases NLRP3 inflammasome activation and activates pyroptosis in ILICI, while hepatocyte-specific knockdown attenuates inflammasome activation

Since the knockdown of caspase-3 in the entire liver cell population worsened liver injury (Fig. 4C–E), we considered whether we were activating necroptosis, which occurs when apoptosis is blocked. We looked for the expression of necroptosis pathway proteins in the liver lysates of C3-ASO + ICI-treated mice (Sup. Fig. 3B). No pMLKL was detected with caspase-3 knockdown, ruling out a switch to necroptosis. We again noted an increase in total RIPK3 protein with ICIs which corresponds to an increase in inflammatory cells [24]. We then turned our attention to the pyroptosis pathway. Unconjugated C3-ASO + ICI-treated livers displayed upregulation of NLRP3, ASC, IL-18, and cleaved caspase-11, as well as uptrending cleaved caspase-1 (Fig. 5G). Importantly, unconjugated C3-ASO + ICI-treated livers clearly displayed a statistically significant increase in the production of GSDMD-N, indicating pyroptotic cell death was occurring in these livers. These findings suggest that total liver knockdown of caspase-3 likely worsened liver injury by promoting pyroptosis in the liver. As total liver caspase-3 knockdown worsened liver injury we hypothesized that preventing apoptosis in the non-parenchymal immune cells results in increased cytokine production and promotion of inflammation driving further liver injury. We stained livers for Annexin V and did not detect a difference between C3-ASO + ICI and CTRL-ASO + ICI-treated mice (data not shown), likely due to the fact that Annexin V stains cells undergoing both apoptosis and pyroptosis [30]. Next, we stained livers of C3-ASO + ICI or CTRL-ASO + ICI-treated mice for CD68 and quantified random fields by the ImageJ IHC Profiler Plugin and indeed found significantly more CD68 staining cells present in the C3-ASO-treated livers (Sup. Fig. 3C, D).

ICI treatment in GalNac-C3-ASO mice did cause detectable ASC, NLRP3, cleaved caspase-11, and IL-18 when compared to IgG-treated controls. However, in comparison to the GalNac-CTRL-ASO + ICI group, mice with hepatocyte-specific caspase-3 knockdown (GalNac-C3-ASO) treated with ICI, displayed attenuated NLRP3 activation, less protein expression of downstream mediators, and no GSDMD-N (Fig. 5G). Therefore, hepatocyte-specific caspase-3 knockdown attenuates NLRP3 inflammasome activation in ICI-treated mice.

### Single-cell resolution phenotyping of livers of ICI-treated mice reveals multiple clusters of innate and adaptive immune cells

To better characterize the spatial and phenotypic features of cells involved in ILICI, we utilized a novel and powerful multiplex imaging platform known as IMC which allows for the staining of formalin-fixed paraffin-embedded (FFPE) tissues with a cocktail of up to 40 different antibodies simultaneously [31]. Each antibody is conjugated to a unique metal isotope with a distinct mass. Laser ablation of a region of interest (ROI) of stained tissue in 1 µm increment releases metal material which is then ionized and travels toward the detector according to its mass (Sup. Fig. 4A). This generates highly spatially resolved signal intensities, which can then be used to directly visualize cellular composition and architecture and to extract single-cell resolution data. While IMC is being used in human research, to our knowledge, it has not been previously used on mouse liver. Therefore, we developed, tested, and validated a mouse-specific IMC antibody panel. This consisted of 35 markers (Sup. Fig. 4B) tailored to the delineation of hepatic structure, immune composition, and activation of pathways and molecules of interest (NLRP3 and cC3).

**Fig. 6:**
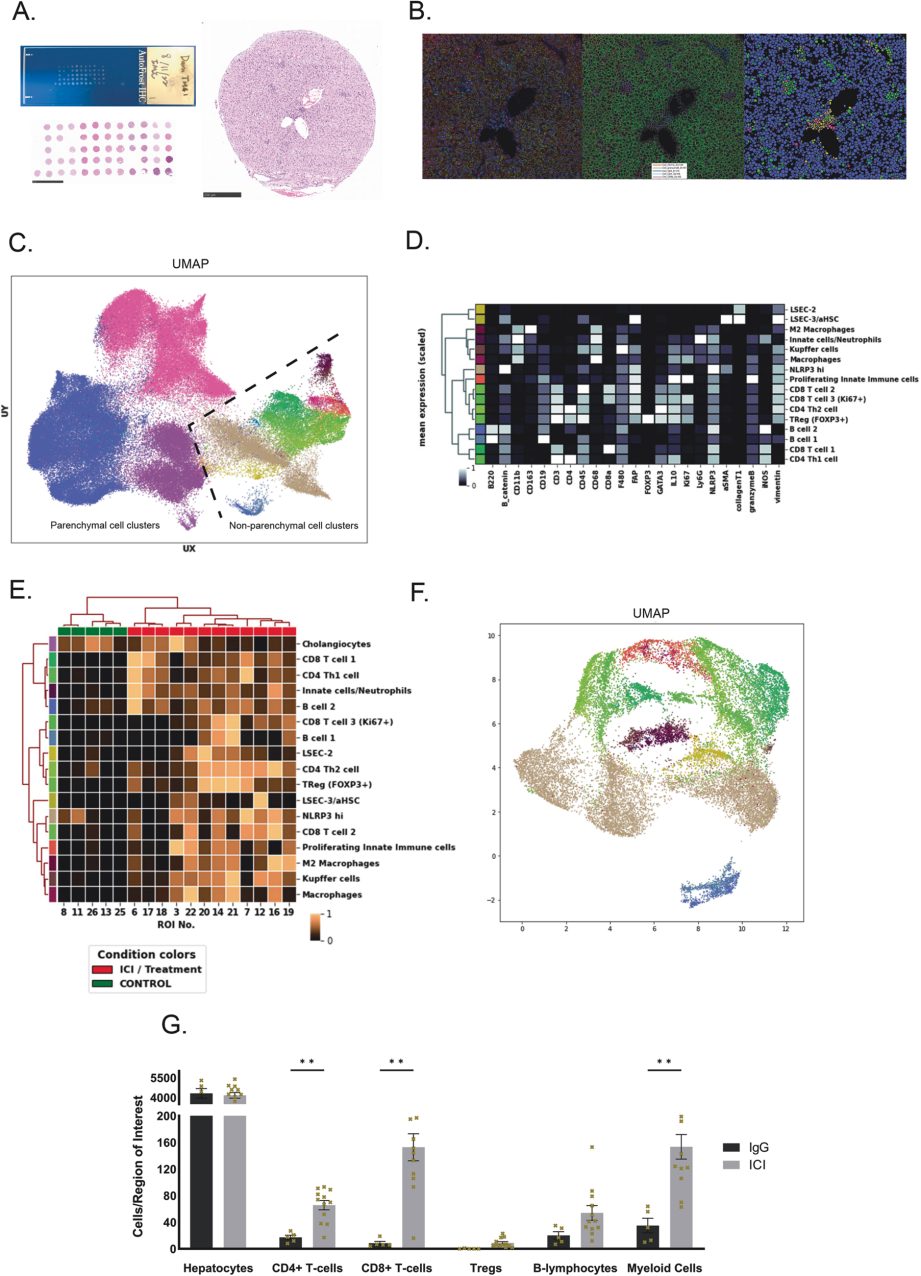
Single-cell resolution phenotyping of livers of ICI-treated mice using Imaging Mass Cytometry (IMC). **A** (left) Tissue microarray (TMA) with livers of control and ICI-treated CTLA4^+/-^ mice. The corresponding H&E-stained TMA section is shown. (right) H&E-stained section of one region of interest (ROI) from ICI-treated liver. Scale bar = 250 µm. **B** Pseudocolored spatial rendering of acquired IMC data from ICI-treated liver showing (left) multi-channel signal, (center) single-cell segmentation mask, and (right) single-cell spatial and phenotypic identity. **C** Single-cell clustering and phenotypic signatures were visualized using pseudocolored uniform manifold approximation and projection (UMAP) using all ROIs. **D** Scaled mean marker expression heatmap showing marker signatures for all non-parenchymal phenotypic clusters. **E** Cluster abundance heatmap showing the relative abundance of non-hepatocyte clusters in control (green) and ICI-treated (red) ROIs. **F** UMAP of the non-parenchymal cell (NPC) clusters only. **G** Quantification of number of cells/ROI in IgG and ICI-treated livers. Data are mean ± SEM. *n* = 5 IgG, 12 ICIs ROIs, from 4 IgG mice and 8 ICI mice. *P*-values were calculated using multiple unpaired *t*-tests. *P*-value **(<0.01).

We then generated a tissue microarray (TMA) consisting of FFPE liver sections from IgG control and ICI-treated CTLA4^+/-^ mice, and stained two serial sections, one with H&E and one with our antibody cocktail (Fig. 6A). After laser ablation we were able to visualize localization of signal for all markers across ROIs, and generate a segmentation mask using marker topology to outline and extract single-cell data (Fig. 6B) [32].

With the single-cell data, we used the Phenograph algorithm (see methods) to stochastically segregate all cells into data-driven phenotypic clusters (Fig. 6B–D) [33]. In total, cells were associated with 22 distinct phenotypic clusters. Interestingly, among the 6 hepatocyte clusters identified, one relatively small cluster bore the marker signature of activated apoptosis (cC3 + ). Individual cells were visualized on each ROI colored according to their cluster phenotype (Fig. 6B), and all clusters were compared across all ROIs using dimension-reducing representations (Fig. 6C) [34].

We identified 16 clusters comprised of immune and non-parenchymal cells according to their unique marker expression signatures (Fig. 6D). Of these, 8 corresponded to adaptive immune cells, 2 to LSECs mixed with some stellate cells, and 6 were innate immune cells (Fig. 6D). Focusing on the NPC compartment, we compared the relative abundance of each NPC cluster across all IgG and ICI-treated ROIs (Fig. 6E), and again visualized similarities and differences across all NPC clusters using dimension-reducing techniques (Fig. 6F). This revealed distinct co-occurrences and groupings of NPC clusters based on treatment condition. In quantifying the relative abundances of phenotypic clusters in isotype control and ICI-treated livers, as expected we observed significantly more CD4 + T-cells, CD8 + T-cells, and myeloid cells in ICI-treated livers compared to IgG-treated control (Fig. 6G).

### Spatial analysis of single-cell IMC data reveals a direct interplay between the adaptive and innate immune cells in the livers of ICI-treated mice

**Fig. 7:**
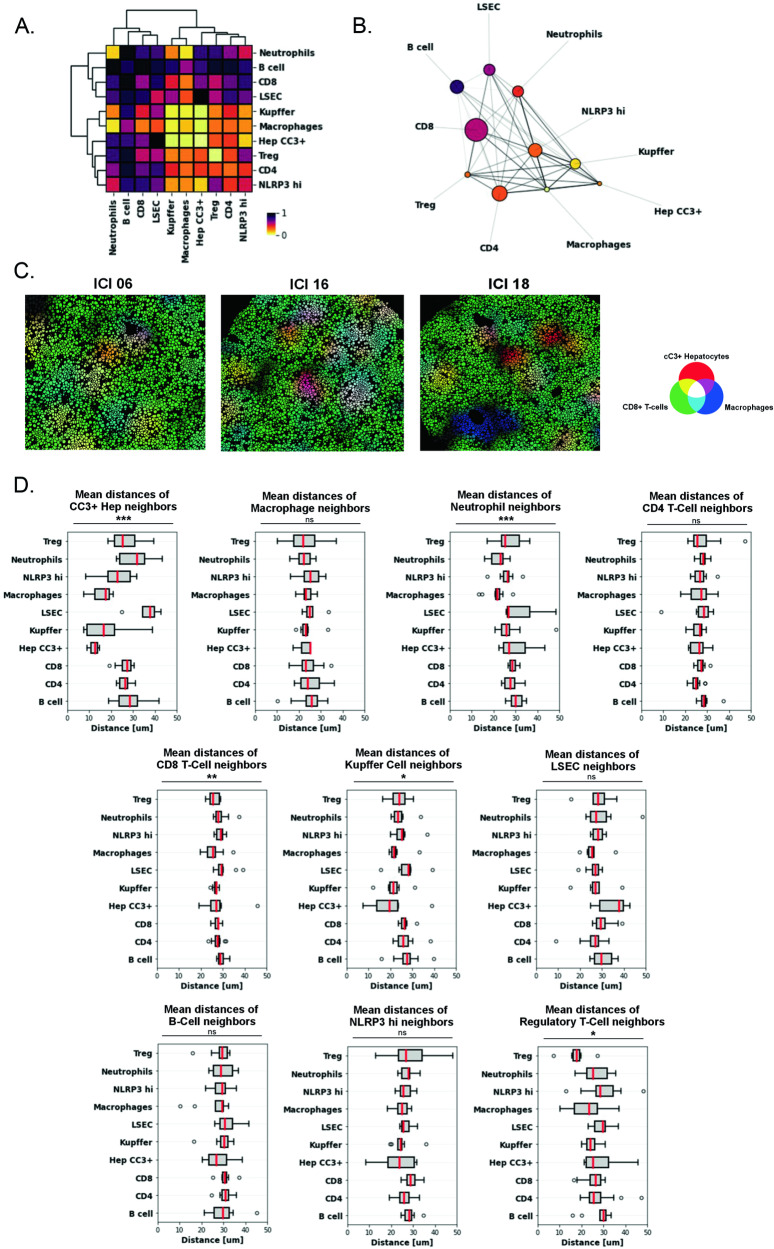
Spatial analysis of single-cell IMC data reveals a direct interplay between the adaptive and innate immune cells with apoptotic hepatocytes in ILICI. **A** Heatmap depicting pairwise distance rank between NPC clusters, cleaved Caspase-3+ (CC3 + ) hepatocytes included. Scale: 1/black: furthest distance pair, 0/yellow: closest distance pair. **B** Feature map summating pairwise distance ranks and spatial distribution of NPC clusters and cC3+ hepatocytes. Node size: abundance, edge opacity: pairwise distance rank. Node pseudocoloring as in Fig. 7A. **C** Pseudocolored single-cell renderings of ICI-treated ROIs depicting spatial interaction zones. Cells colored according to the Red-Green-Blue channel composite based on spatial interaction with cC3+ hepatocytes (red), CD8 + T-cells (green), and macrophages (blue). White/gray coloring denotes the interaction zone consisting of all three cell types. **D** Plots of mean distances between cells of a given phenotype and neighboring cells of other phenotypes across *n* = 12 ICI-treated ROIs. Data are mean with range. *P*-values were calculated using Welch’s ANOVA. *P*-value *(<0.05), **(<0.01), ***(<0.001).

IMC preserves the spatial architecture of ablated tissues, allowing for input of single-cell data into advanced computational methods aimed at resolving spatial interactions amongst different cell clusters. Focusing on the spatial relationships between LSECs, 4 lymphocyte clusters (CD4, CD8, Tregs, B cells), 4 myeloid clusters (Kupffer, macrophage, Neutrophils, NLRP3 hi myeloid cells), and the cC3+ hepatocyte cluster (Hep cC3 + ), we included the 50 nearest neighbors of each cell and computed the average distance of every cell to every phenotype and subsequently ranked the distances between all cluster pair combinations in ICI-treated ROIs as shown in Fig. 7A. We determined that the closest pairwise interactions with cC3+ hepatocytes belonged to Kupffer cells, macrophages, and the NLRP3 hi cluster (Fig. 7A). Stochastic grouping of pairwise distance ranks shows a block of distinctly close interactions amongst Kupffer cells, macrophages, cC3+ hepatocytes, Tregs, CD4 + T-cells, and NLRP3-high cells. A feature map summating pairwise distance ranks with reduced dimensionality again highlights high interactivity and spatial clustering between cC3+ hepatocytes and these immune cells (Fig. 7B). CD8 + T-cells display intermediate interactivity with cC3+ hepatocytes, however, this is likely skewed by the relatively high abundance of CD8 + T-cells throughout the ROIs, which includes far non-interacting cells. Regardless, it is clear that the immune cells in closest proximity to cC3+ dying hepatocytes are of myeloid origin.

To directly visualize spatial interactions of clusters in ROIs, we generated interaction zones of segmented cells that are pseudocolored according to composite Red-Green-Blue channel intensities determined by a given cell’s proximity to three markers (proximity to cC3+ hepatocytes (red), CD8 + T-cells (green), and macrophages (blue)) (Fig. 7C). Here we observed several patterns of cellular interaction, including zones of cC3+ hepatocyte & CD8 + T-cell interaction (yellow), CD8 + T-cell & macrophage interaction (teal), cC3+ hepatocyte & macrophage interaction (violet), and cC3+ hepatocyte, CD8 + T-cell & macrophage interaction (white-gray). By mean distance, Kupffer cells, macrophages, and NLRP3hi myeloid cells were significantly closer neighbors of cC3+ hepatocytes than other cell types (Fig. 7D). Among neighbors of CD8 + T-cells, macrophages were significantly closer than all other phenotypes. Significant differences were also observed in the spatial distribution of neighbors of neutrophils, Kupffer cells, and Tregs (Fig. 7D).

**Fig. 8:**
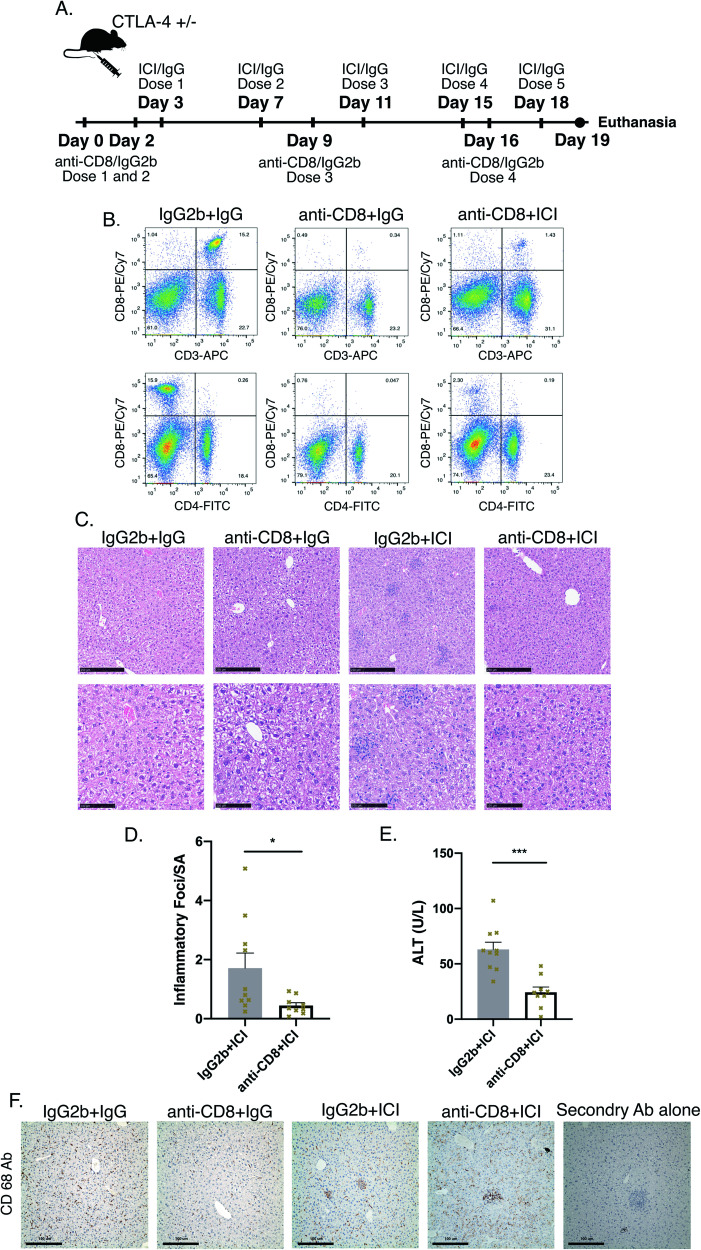
Depletion of CD8 + T-cells in ILICI model protects. **A** Murine model of anti-CD8 CTL depletion. **B** FACS Analysis. **C** Representative H&E images at 10X and 20X magnification. **D** Inflammatory Foci/surface area n = 10 IgG2b+ICI and 9 anti-CD8 + ICI from two independent experiments. **E** ALT *n* = 10 IgG2b+ICI and 9 anti-CD8 + ICI. **F** Representative IHC for CD68. *P*-values were calculated using a *t*-test. *P*-value *(<0.05), ***(<0.001).

Given the close proximity of CD8 + T-cells to cC3 hepatocytes and their known role in cytotoxicity, we tested the effect of depletion of these cells in our ILICI model using injections of a monoclonal antibody specific to murine CD8. After two injections of anti-CD8 or isotype control IgG we again treated mice with ICIs or isotype control IgGs for five injections (Fig. 8A). CD8 depletion by itself did not cause any liver injury or elevated liver enzymes (not shown), and confirmed depletion by FACS (Fig. 8B). Interestingly, CD8 depletion significantly protected against liver injury (Fig. 8C–E). However, a few foci of necroinflammation remained after treatment with ICI, which stained strongly for CD68, a macrophage marker (Fig. 8F). These findings suggest that CD8 + T-cells are principal effectors of liver injury and cell death in ILICI.

Overall our results demonstrate direct spatial crosstalk between cells of the adaptive and innate immune systems in the livers of ICI-treated mice. Furthermore, adaptive and innate immune cells each spatially interact with apoptotic hepatocytes. Lastly, we were able to observe zones of three-way interaction between adaptive immune cells, innate immune cells, and cC3+ hepatocytes, evidence that the interplay of adaptive and innate immunity plays a significant role in hepatocyte death in ILICI.

## Discussion

High-grade hepatotoxicity from immunotherapy is a significant barrier to the use of these life-saving medications in cancer patients. Understanding the underlying mechanisms of ILICI is necessary to uncouple unwanted liver injury from the desired anti-tumor response. Here we report a novel mouse model of ILICI using a combination of two ICIs in CTLA4^+/-^ mice. CTLA4^+/-^ mice have no baseline phenotype but developed more severe and consistent hepatotoxicity in response to ILICI making them a suitable preclinical model to study this disease. To understand how hepatocytes die we probed the apoptotic, pyroptotic, and necroptotic cell death pathways. We detected cleavage of caspase-3, the executioner of apoptosis in hepatocytes of ICI-treated mice. Conversely, necroptosis was not occurring as we could not detect pMLKL activation. Interestingly, ICI treatment resulted in the activation of the NLRP3 inflammasome and the production of pro-inflammatory cytokines such as IL-6, IL-1β, IL-18, and Tumor necrosis factor (TNF).

Since we detected apoptosis of hepatocytes, we tested whether knocking down caspase-3 protects from ILICI. Capsase-3 knockdown using unconjugated ASO that accumulates in the liver and affects both hepatocytes and NPCs actually worsened liver injury in ICI-treated mice and resulted in further induction of NLRP3, GSDMD-N, and pyroptotic cell death. Interestingly, during apoptosis, caspase-3 blocks pyroptosis by cleaving GSDMD at a distinct site that inactivates the protein [35]. Therefore, since both the inflammasome pathway and apoptosis are active in our model, it may be that by blocking apoptosis in macrophages and other immune cells while treating with a CTLA4 and PD-L1 inhibitor, we enhance pyroptosis.

Pyroptosis is a highly inflammatory form of death leading to the release of inflammatory mediators into the liver milieu, worsening liver injury. In blocking apoptosis in the immune and NPC compartments, it is possible we immortalized a contingent of CTLs and myeloid cells already contributing to inflammatory injury, further fueling unchecked inflammation. Clayton et al. demonstrated that CTL-mediated killing of macrophages is caspase-3 dependent [16]. Additionally, inefficient killing of macrophages by CTLs has been shown to result in hypersecretion of Interferon (IFN)γ and pro-inflammatory chemokines [16]. Therefore, we hypothesized that impaired macrophage clearance contributes to the augmented injury with total liver caspase-3 knockdown in our model. To test this we stained livers of C3-ASO + ICI and CTRL-ASO + ICI-treated mice for Annexin V but could not detect a difference. Although apoptosis was blocked in C3-ASO + ICI-treated livers, pyroptosis was increased and both apoptotic and pyroptotic cells stained positive for Annexin V [30]. Our IHC staining for CD68 does show significantly stronger staining macrophage infiltrates in the livers of C3-ASO + ICI-treated mice. However, these livers also displayed more injury and the increase in CD68 staining may reflect this. Further investigation into the temporal behavior and phenotype of these cells is necessary to better interpret our findings and determine whether they play a contributory or reactionary role in liver injury in the C3-ASO ICI mice.

Given the worsening of injury with total liver caspase-3 knockdown, we next attempted a hepatocyte-specific approach, using GalNac-conjugated ASO that preferentially accumulates in hepatocytes [29]. This approach partially protected against liver injury and did not result in the activation of pyroptosis we had detected with whole liver knockdown. Interestingly, preventing hepatocyte apoptosis also decreased NLRP3 activation suggesting an inflammatory feedback loop where the release of DAMPs by dying hepatocytes further fuels the NLRP3 inflammasome, propagating inflammation. Additional experiments using genetic approaches such as adult hepatocyte-specific knockout of caspase-3 in combination with blocking other caspases in hepatocytes will be informative and necessary to confirm findings.

While ICIs were primarily developed to combat T-cell exhaustion and activate CTL responses in tumors, they also affect innate immune cells [3, 5, 36]. An important, clinically relevant, and novel aspect of our work is the discovery of the role of the NLRP3 inflammasome in ILICI. The upregulation of NLRP3 in our model, which is aggravated by caspase-3 knockdown, offers a mechanism by which innate immune cells participate in liver injury. We observed NLRP3 staining in NPCs and scattered hepatocytes abutting areas of injury. Further studies are needed to study the NLRP3 inflammasome in the liver as a therapeutic target in ILICI and to assess if a liver-targeted approach to blocking NLRP3 could treat ILICI and allow for immunotherapy continuation in high-grade hepatotoxicity.

To characterize the subsets of immune cells participating in necro-inflammatory foci and their relation to hepatocyte apoptosis, we applied IMC, a cutting-edge immune-phenotyping technique that allows the characterization of spatial dynamics among cells [31]. To our knowledge, this is the first reported use of IMC in murine liver. We designed our panel with cell death effectors and proteins of interest such as cC3 and NLRP3 in mind. We identified 6 parenchymal clusters and 16 clusters of NPCs. We show that ICI treatment caused significant liver injury and infiltration of adaptive and innate immune cells in areas containing cC3+ hepatocytes. Indeed, spatial analysis of single-cell data found myeloid cells as the nearest neighbors of the cC3+ hepatocytes, demonstrating a direct interaction. Our work is not without limitations, one of which is the lack of cancer in the ICI-treated mice. Future work will need to focus on building a tumor model in the CTLA4 + /- mice treated with ICIs to better recapitulate human disease. In addition, performing IMC on mice is a novel technology and is limited by the availability of validated murine antibodies. We were unable to measure pyroptosis as there were no suitable antibodies to detect cleaved GSDMD-N in mouse tissue. Future work using larger antibody panels and using human liver tissue will need to be done to identify the subtypes of immune cells present and their role in liver inflammation.

The dysregulation of adaptive and innate immunity caused by ICIs induces a pro-inflammatory state in the liver that hyperactivates both CTLs and myeloid cells. This inflammatory state drives the recruitment of more immune cells to a milieu already engulfed in flames. Since the interaction of macrophages and CD8 T-cells appears central to hepatocyte cell death, we used an anti-CD8 antibody to deplete these cells prior to ICI treatment. This resulted in marked but not complete protection from ILICI, with a few foci of injury-containing macrophages persisting in the liver. The effect of ICIs on innate immunity is very under-studied, but it has been shown that ICIs promote pro-inflammatory M1 polarization in macrophages [37]. There is also evidence that macrophage activation plays a role in human patients with ILICI [38]. Taken altogether, we propose that macrophages are the initiators of injury, attracting T-cells to the liver and orchestrating the off-target hepatotoxicity of ICIs. We did not attempt macrophage depletion within the scope of this study and acknowledge that future studies using genetic models to knockout and manipulate macrophages are necessary to confirm our findings.

To our knowledge, this is the first report of NLRP3 inflammasome activation in ILICI, and the first to propose a mechanism by which innate immune cells participate in liver injury. This work helps inform future therapeutic approaches targeting inflammation and cell death in a liver-specific manner, which will be critical in treating hepatotoxicity from immunotherapy without affecting the anti-tumor response. In summary, we show that in ILICI the disruption of adaptive and innate immunotolerance in the liver is synergistically coupled to the activation of the NLRP3 inflammasome, leading to an unchecked inflammatory state which leads to hepatocyte apoptosis, a perfect storm of cell death.

## Materials and Methods

### Animals

Haploinsufficient CTLA4^+/-^ C57BL/6N mice [39], and MLKL^-/-^ C57BL/6 mice (provided by Professor Warren Alexander, Walter and Eliza Hall Institute of Medical Research, Parkville, Australia) were housed under standard laboratory conditions in an environmentally controlled room with a 12 h light/dark cycle with unrestricted access to appropriate food and water. The experimental protocol was approved by the Institutional Animal Care and Use Committee at USC. All the animals received humane care in accordance with the outlined criteria in the Guide for the Care and Use of Laboratory Animals prepared by the National Academy of Sciences.

### In vivo experiments

We generated our injury model using CTLA4 haploinsufficient mice that have no baseline phenotype but lack one CTLA4 allele rendering them more susceptible to autoimmunity [39]. CTLA4^-/-^ knockout mice develop a florid multi-organ lymphoproliferative disease that is lethal by 3 weeks of age and cannot be used. 8–12 week-old CTLA4^+/-^ littermates (male and female) were randomized to either ICI treatment with murine anti-CTLA4 (BioXcell, Lebanon, NH, USA, 9D9 clone, 10 mg/kg) + murine anti-PDL1 (Genentech, South San Francisco, CA, USA, 10 mg/kg, provided by Dr. Ira Mellman) or isotype control treatment with murine IgG1α (BioLegend, San Diego, CA, USA, 10 mg/kg) + murine IgG2β (BioXcell, 10 mg/kg). Doses were determined based on previously published data [40–42] and dose-response experiments in our lab. Treatments were administered via intraperitoneal injection for 1, 2, and 4 weeks, and after analyzing data we decided 2 weeks was sufficient for the model. We administered 5 doses over a 14-day period (Day 0, 3, 6, 9, 12). At the end of the treatment period, mice were euthanized humanely, and blood and tissue samples were harvested and processed. Data are from at least 4 independent experiments; “n” denoted in figures or figure legends indicates biological replicates for each experimental group/condition, except when indicated as regions of interest or ROIs in IMC experiments. Each experiment was repeated at least four times unless indicated. Littermates were used and always randomized to treatment and control groups in an unblinded fashion during in vivo experiments. Since crosses between CTLA4 + /+ and CTLA4 + /- breeding pairs produce about 50% CTLA4 + /- offspring multiple breeding pairs were generated to have sufficient numbers of mice. The sample sizes were based on an available number of littermates that were 10-12 weeks of age and CTLA4 + /- at the time of the experiment. Animals that were excluded from the analysis include those that died prior to the treatment endpoint or had significant internal hemorrhage.

### Antisense oligonucleotide (ASO) treatment

Antisense oligonucleotide targeting mouse Caspase-3, and control scrambled oligonucleotide (CTRL-ASO) were provided by Dr. Sue Murray, Ionis Pharmaceuticals (Carlsbad, CA, USA). The oligonucleotides were synthesized as 20-nt uniform chimeras containing five nuclease-resistant 2′-O-methoxyethylribose-modified phosphonothioate residues on the 5′ and 3′-ends, flanking a 2′-deoxyribonucleotide/phosphonothioate region, which supported RNase H1-based cleavage of the targeted mRNA. The nucleotide sequences for Caspase-3-ASO and control scrambled ASO were ‘CTGCGTCCACATCCGTACCA’ and ‘CCTTCCCTGAAGGTTCCTCC’, respectively. For hepatocyte-targeting ASO, we used N-acetyl galactosamine (GalNac) conjugated moieties which are preferentially taken up by asialoglycoprotein receptor-expressing hepatocytes and can be delivered at much lower doses without affecting potency [29].

In addition to treatment with IgG vs ICI, littermate mice were randomized to concurrent treatment with either unconjugated control scrambled ASO (Ionis, 50 mg/kg), unconjugated C3-ASO (Ionis, 50 mg/kg), GalNac-conjugated control scrambled ASO (Ionis, 1 mg/kg), and GalNac-conjugated C3-ASO (Ionis, 1 mg/kg). ASO treatments were administered via intraperitoneal injection, with 5 administrations over a 14-day period. ASO treatment was initiated before IgG or ICI treatment so as to administer two ASO doses before the first IgG or ICI dose, after which IgG/ICI and ASO injections were alternated. The total treatment period when combining ASO and IgG/ICI administrations was 21 days.

### Serological Studies

Blood samples were collected into microtainer tubes with clotting and serum separating gel (BD, Franklin Lakes, NJ, USA), allowed to clot, and serum was separated by centrifugation at 7000 rpm (x10 min at 4 °C). Serum concentrations of alanine aminotransferase (ALT) were assessed by spectrophotometric assay (FLUOstar Omega by BMG Labtech, Ortenberg, Germany) using an ALT measuring kit according to manufacturer’s instructions (TECO Diagnostics, Anaheim, CA, USA). Serum concentrations of chemokines were assessed on the Luminex xMAP platform, using the *Milliplex High Sensitivity T Cell Panel Immunology Multiplex Assay* kit according to manufacturer’s instructions (Cat# MHSTCMAG-70K, Millipore-Sigma, Burlington, MA, USA). Undetectable and out-of-range (OOR) values could not be included.

### Histological analysis

For histological analysis, livers were harvested and fixed with 10% neutral buffered formalin for 24 h. The fixed livers were then embedded in paraffin and cut into 5 μm-thick sections at the Research Center for Liver Disease Histology and Cell Imaging Core. Hematoxylin-eosin (H&E) staining was performed on all specimens, and the sections were evaluated under light microscopy. Histological grading and characterization were performed by a liver pathologist (G.K), who was blinded to the treatment group of all analyzed samples. H&E-stained liver sections were digitally scanned at 20x magnification. Quantification of inflammation was performed blinded to condition in ImageJ (National Institute of Health, Bethesda, MD USA) using H&E digital scans by counting the number of inflammatory foci throughout the entire stained liver section. The number of foci counted was then divided by the surface area (in pixels) of the liver section as measured by ImageJ.

### Immunohistochemistry (IHC)

Immunostainings for CD4, CD8, F4/80, and cleaved Caspase-3 were performed on tissue sections of formalin-fixed paraffin-embedded (FFPE) livers from IgG and ICI-treated CTLA4^+/-^ mice. Antibodies used were anti-CD4 (1:200, Cat#: AB183685, Abcam, Cambridge, UK), anti-CD8 (1:100, Cat# 14-0808, EBioscience, San Diego, CA, USA), anti-F4/80 (1:200, Cat#: D2S9R, Cell Signaling Technology, Danvers, MA, USA), anti-CD68 (1:300, Cat#97778, Cell Signaling Technology, Danvers, MA, USA), and anti-cleaved Caspase-3 (1:1000, Cat#: 9664, Cell Signaling Technology). The Leica IHC automated stainer (Bond III; Leica Biosystems, Wetzlar, Germany) was used at the USC pathology laboratory (cleaved caspase-3) and UCLA pathology (CD4, CD8, F4/80). All slides were sectioned on the same day and IHC experiments were done simultaneously to control for batch variability. IHC intensity was measured using the ImageJ IHC Profiler Plugin, as previously described [43].

### Immunofluorescence

Immunofluorescence staining was performed on tissue sections of formalin-fixed paraffin-embedded livers from IgG and ICI-treated CTLA4^+/-^ mice. Briefly, a water bath was preheated to 85 °C, and glass jars covered with aluminum and plastic covers were placed in the water. Unstained tissue sections were dewaxed three times for 5 min each using fresh xylene in a fume hood. The slides were then hydrated through an alcohol gradient, starting with 2×100% ethanol (Koptec, King of Prussia, PA, USA) for 5 min, followed by 2 × 95% ethanol for 5 min, 1×70% ethanol for 5 min, and then 1x DPBS (VWR, Radnor, PA, USA) for 5 min. The Na-Citrate buffer (Sigma-Aldrich, St Louis, MO, USA) was preheated to just boiling in the microwave, then the glass container with slides and buffer was placed in a water bath set at 95 °C for antigen retrieval for 30 min, ensuring the water bath and buffer temperature was maintained above 80 °C. The slides were then washed with PBS for 5 min, and if necessary, endogenous peroxidase activity was blocked with 1% H_2_O_2_ in DPBS for 20 min. The tissue was permeabilized with 0.3% tween-20 (Bio-Rad, Hercules, CA, USA) for 45 min at 37 °C, then blocked with 50% seablock (Thermo Scientific, Waltham, MA, USA) + 50% 1x PBS + 0.3 M glycine. Primary antibody tubes were prepared with cleaved caspase-3 Rabbit (1:2000, cat# 9664, Cell Signaling Technology), NLRP3 Rat (20 µg/mL, cat# 7578, R&D Systems, Minneapolis, MN, USA), CD45 Goat (10 µg/mL, cat# AF114, R&D Systems), HNF-4α Mouse (1:150, cat# NBP2-45989, Novus Biologicals, Minneapolis, MN, USA), and permeabilized slides were incubated with primary antibody overnight at 4 °C. Incubated slides were washed 3x with PBS and then dried. Secondary antibody tubes were prepared with recommended dilutions in DPBS. The slides were then incubated with secondary antibodies and mounted using Immunogold + DAPI (50 µL, cat# P36931, Invitrogen, Waltham, MA, USA), then imaged by confocal microscopy (Leica Biosystems).

### Western blot

Liver and spleen lysates were prepared by dounce homogenization of 80 mg fresh liver or spleen tissue in 1 mL radioimmunoprecipitation assay (RIPA) buffer with added protease inhibitor and phosphatase inhibitor cocktails (Sigma-Aldrich) as previously described [24]. For testing the specificity of GalNac knockdown, GalNAc-Control-ASO and GalNac-C3-ASO-treated livers underwent collagenase perfusion to isolate the non-parenchymal cell (NPC) and hepatocyte compartments prior to homogenization as previously described [24]. Supernatant protein concentration was measured using a Bradford protein spectrophotometric assay kit (Bio-Rad) followed by Western Blotting as previously described [24]. The membranes were then incubated with the desired primary antibodies overnight followed by secondary antibody (Cell Signaling Technology) for 1 h. Finally, the proteins were visualized using Luminol enhanced chemiluminescent (ECL) reagent (Cat# 34095, Thermo Fisher Scientific) on a ChemiDoc MP Imaging System (Bio-Rad). Image analysis, densitometry, and gray level calculation for each band in pixels normalized to a reference band (depicted under WB images) was performed using Image Lab Software with background subtracted per the Bio-Rad manual. Full-length original western blots are available in the supplemental materials section.

### Imaging mass cytometry (IMC) antibody staining

We designed an IMC metal-conjugated antibody panel of 35 markers and tested this on control liver and spleen tissue (Sup. Fig. 4B). We then constructed a tissue microarray (TMA) consisting of 1 mm diameter cores from FFPE livers of isotype control and ICI-treated CTLA4^+/-^ mice and positive controls for antibody verification (spleen and CTLA4 KO livers). A TMA section slide was heated at 60^o^C for 90 min and subsequently immersed in xylene for 20 mins. The slide then underwent the following ethanol washing steps, 5 min each; 100%, 95%, 80%, and 70%. After washing, the TMA slide was immersed in Tris-EDTA antigen retrieval solution for 30 min at 95 ^o^C and was left to cool inside the solution for 30 min at room temperature. The slide was blocked with 3% BSA for 45 min and then was stained overnight with our mouse antibody panel at 4^ o^C. The next day the stained TMA slide was washed twice with PBS-0.1% Triton X solution and 1x PBS for 8 min each. The slide was then incubated with 191 Iridium, a nuclear stain, for 40 min, washed with dH_2_O, and dried off prior to laser ablation.

### IMC laser ablation, image capturing, and processing

Laser ablation and image acquisition were performed using the Hyperion Imaging System (Standard BioTools, previously Fluidigm, South San Francisco, CA, USA) following the manufacturer’s instructions. Regions of interest (ROIs) were selected according to H&E-stained serial sections of our TMA. ROIs were laser ablated and signal data was recorded by proprietary commercial software (Standard BioTools). Overall, 5 isotype control ROIs and 12 ICI-treated ROIs were ablated for analysis. An additional 9 ROIs consisting of spleen and positive controls (CTLA4 KO livers) were ablated at the same time for antibody validation and segmentation model training but excluded from analysis. Each ROI measured roughly 700 µm x 700 µm in size. Using MCD Viewer software (Standard BioTools), all ROIs were reviewed for signal quality across all markers. Converted TIFF images of each channel (marker) across all ROIs were used to execute a cell segmentation program and extract single-cell data as described in Greenwald et al. [32]. Across the 17 ROIs of interest, a total of 77 692 single cells and their individual marker expressions were extracted for downstream analysis.

### Cell clustering and spatial analysis

The 77 692 segmented cells, each bearing signal data for 32 selected markers (excluding nuclear markers), were input into the Phenograph algorithm as in Levine et al.[33] In a stochastic and data-driven manner, the Phenograph program arranges and clusters all cells according to their phenotypic similarity, as determined by marker expression. In this analysis, Phenograph assigned all cells to one of 22 phenotypic clusters. Further sub-clustering was performed on select markers according to the cellular compartment of interest. Visualization of single-cell data and clustering using uniform manifold approximation and projection (UMAP) was performed as in Becht et al. [34].

For spatial analysis, we focused primarily on cells from non-parenchymal clusters and the cC3+ hepatocyte cluster. To perform pairwise distance ranking, we began by taking the first 50 neighbors of each cell, determining their phenotypes, and measuring the average distance of each cell to every phenotype. In this way, we could determine any phenotype’s average distance to any other phenotype within the former’s neighbors (Fig. 7D). In order to obtain a symmetric rank (Fig. 7A) for any given pair, the raw mean distance values (in micrometers) were ranked and scaled to the unit interval, followed by taking the geometric mean of rank(phenotype A, phenotype B) and rank(phenotype B, phenotype A). The resulting symmetric pairwise distance rank (PDR) between select non-parenchymal clusters and cC3+ hepatocytes represents the relative distance between phenotypes when they are in each other’s neighborhood. The feature map plot of cluster pairwise distance ranks (Fig. 7B) was obtained by placing nodes according to a UMAP-embedding of each row vector from Fig. 7A, where edge weights represent the symmetric PDR, while node size and color reflect the average frequency and mean PDR (per row) in the ICI samples. ROI images depicting spatial interaction zones were generated by pseudocoloring segmented cells according to the Red-Green-Blue color model based on spatial interaction with cC3+ hepatocytes (red), CD8 + T-cells (green), and macrophages (blue).

### IMC data and code availability

All datasets, notebooks used for analysis, and the respective code will be made available to other researchers upon reasonable request to the corresponding author.

### Antibodies and reagents

In addition to previously mentioned antibodies, the following antibodies were used: Casp3, NLRP3, Casp11, IL-18, IL-1β, ASC, pMLKL (1:1000 dilution, Cell Signaling Technology), RIPK1 XP (1:4000 dilution, Cell Signaling Technology), GAPDH (1:16000 dilution, Cell Signaling Technology), Casp1, GSDMD (1:1000 dilution, Abcam), and monoclonal RIPK3 antibody provided by Dr. Kim Newton (1:1000, Genentech).

### In vivo CD8 T-cell depletion

We performed in vivo CD8 + T-cell depletion studies using intraperitoneal (IP) injection of monoclonal anti-mouse CD8 (YTS 169.4, BioXCell, Lebanon, NH, USA) in addition to treatment with either IgG or ICI. IP injections of anti-CD8 (BioXCell, 300 µg/animal) or IgG2b (BioXcell, 300 µg/animal) were performed at day 0 and day 3 prior to IgG1 and 2 (IgG) or anti-CTLA4 and anti-PD-L1 (ICI) injections, and once a week thereafter. The four treatment groups in this experiment were anti-CD8 + ICI, anti-CD8 + IgG, IgG2b + ICI, and IgG2b + IgG. CD8 + T-cell depletion was confirmed by FACS analysis of primary splenocytes of treated mice.

### FACS analysis

The depletion of CD8 + T-cell subsets in vivo was confirmed by FACS analysis of primary splenocytes isolated from treated mice. For flow cytometry (FC) analysis we used monoclonal antibodies directed against epitopes of CD8 molecules that are different from the injected treatment anti-CD8. Spleens of anti-CD8 and IgG2b treated mice were harvested, then mashed using a homogenizer, resuspended in washing buffer (PBS + 1% FBS + 0.05% NaN3, VWR, Radnor, PA, USA), and passed through a 70-μm pore size cell strainer. Harvested splenocytes were analyzed by FACS using BD LSRFortessa™ X-20 System (New York, USA). Five-color FC analysis was performed after staining splenocytes with PE-anti-mouse CD19 Abs (Cat #:115508, BioLegend, San Diego, CA, USA), FITC-anti-mouse CD4 Abs (Cat #:100405, BioLegend, San Diego, CA, USA), APC anti-mouse CD3 Abs (Cat #:100236, BioLegend, San Diego, CA, USA), PE/Cyanine7-anti-mouse CD8a Abs (Cat #:100721, BioLegend, San Diego, CA, USA) and PerCP/Cyanine5.5-anti-mouse/human CD11b Abs (Cat #:101228, BioLegend, San Diego, CA, USA). 5-10 ×10^5^ cells were incubated with the above antibodies for 40 min at 4 °C in the dark, washed twice in 0.05% NaN3 and 1% FBS in PBS. Data were acquired by FACSDiva software and analyzed by FlowJo software (BD Bioscience, New York, USA).

### Statistics methods

Data were described as mean ± standard error of the mean (SEM), and mean with range. Unpaired Student’s *t*-test was used to compare differences as appropriate. One-way analysis of variance (ANOVA), Welch’s ANOVA, and two-way ANOVA were used to compare group mean differences as appropriate, with post-hoc analysis of multiple comparisons performed with the Sidak test and Tukey Test. Statistical tests were selected based on sample distribution and comparison of variance across groups. Statistical analysis was performed using Prism software version 6 (GraphPad Software, Boston, MA, USA). *P*-value < 0.05 was defined as statistically significant.

### Supplementary information

Supplemental figure legends

Supplementary Figure 1

Supplementary Figure 2

Supplementary Figure 3

Supplementary Figure 4

Authorship change approval

Original full-length Western Blots

Checklist

## Data Availability

All datasets and analytical methods will be made available to other researchers upon reasonable request to the corresponding author.
