# Supplementary material for: Innate and adaptive immune cell interaction drives inflammasome activation and hepatocyte apoptosis in murine liver injury from immune checkpoint inhibitors
Source: Cell Death Dis. 2024 Feb 14;15(2):140. doi: 10.1038/s41419-024-06535-7 (PMC10866933; doi:10.1038/s41419-024-06535-7)
Supplement: Supplementary file 1 — Supplemental figure legends [file 41419_2024_6535_MOESM1_ESM.docx]

**Supplemental Figure 1. CTLA4+/+ mice treated with ICIs develop mild injury. Inflammatory foci from ICI treatment in CTLA4+/- mice are predominantly seen in hepatic zone 2 and contain lymphocytes and macrophages.** (A) Representative Histology of CTLA4 +/+ mice treated with IgG1+2 or ICIs (PD-L1 + CTLA4 inhibitor) at 10X and 20X magnification. (B) Inflammatory Foci/surface area n=8/group. (C) ALT n= 8 group. (D) Analysis of the distribution of inflammatory foci according to hepatic zonation in livers of ICI and ASO treated CTLA4 +/- mice as scored by liver pathology (GK). (E) Immune cell composition in inflammatory foci in livers of ICI and ASO treated mice as reported by liver pathologist (GK). (F) Immunohistochemistry for CD4, CD8, and F4/80 staining. (left) CD4+ helper T-lymphocytes, (middle) CD8+ cytotoxic T-lymphocytes, (right) F4/80+ macrophages. 40x magnification.

**Supplemental Figure 2. Global MLKL genetic knockout is not protective in ILICI.** (A) 8-12 week old MLKL knockout (C57BL/6 background) littermates were randomized to either ICI treatment with anti-CTLA4 plus anti-PD-L1 or isotype control (IgG1 and IgG2) via IP injection as shown. (B) Representative H&E images of livers from MLKL^-/-^ mice treated with (left) isotype controls and (right) combined ICIs. Top: 20x magnification, Bottom: 40x magnification. (C) Quantification of number of inflammatory foci over surface area in liver sections. Data are mean ± SEM. n= 3 IgG, 4 ICI. P value calculated using unpaired t-test. P value ** (<0.01).

**Supplemental Figure 3. Total liver Caspase-3 knockdown using ASO does not cause liver injury on its own and does not induce necroptosis in the livers of ICI treated mice.** (A) Representative H&E images of livers from CTLA4^+/-^ mice treated with ASO alone showing no inflammation or injury at baseline Top: 20x magnification, Bottom: 40x magnification. (B) Representative western blot of whole liver lysates of treated CTLA4^+/-^ mice for RIPK1, RIPK3 and pMLKL. GAPDH shown for loading control. RIPK3 KO was used as internal WB control to show antibody specificity. (C) Quantification of CD68 staining in CTRL-ASO + ICI and C3-ASO + ICI treated livers n=25 10x fields from 5 mice/group. (D) Representative IHC staining for CD68 of treated livers at 10X magnification.

**Supplemental Figure 4. Developing an IMC panel for liver disease in a murine injury model.** (A) IMC workflow schematic. (B) Panel of 35 metal-conjugated antibodies used for IMC staining of livers of isotype control and ICI treated mice.
