# Supplementary figures and images for: Innate and adaptive immune cell interaction drives inflammasome activation and hepatocyte apoptosis in murine liver injury from immune checkpoint inhibitors
Source: Cell Death Dis. 2024 Feb 14;15(2):140. doi: 10.1038/s41419-024-06535-7 (PMC10866933; doi:10.1038/s41419-024-06535-7)

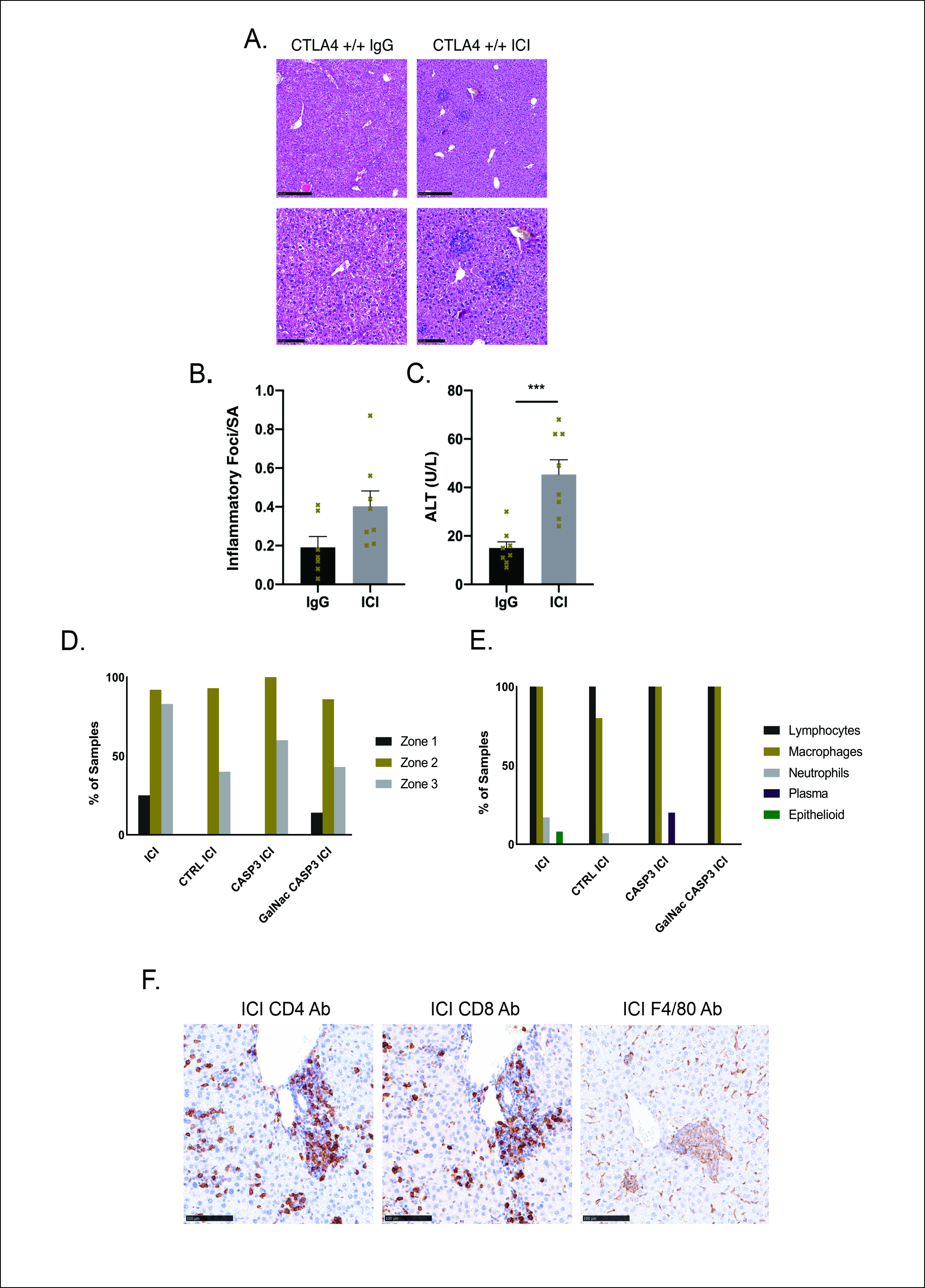

Supplement: Supplementary file 2 — Supplementary Figure 1 [file 41419_2024_6535_MOESM2_ESM.tif]

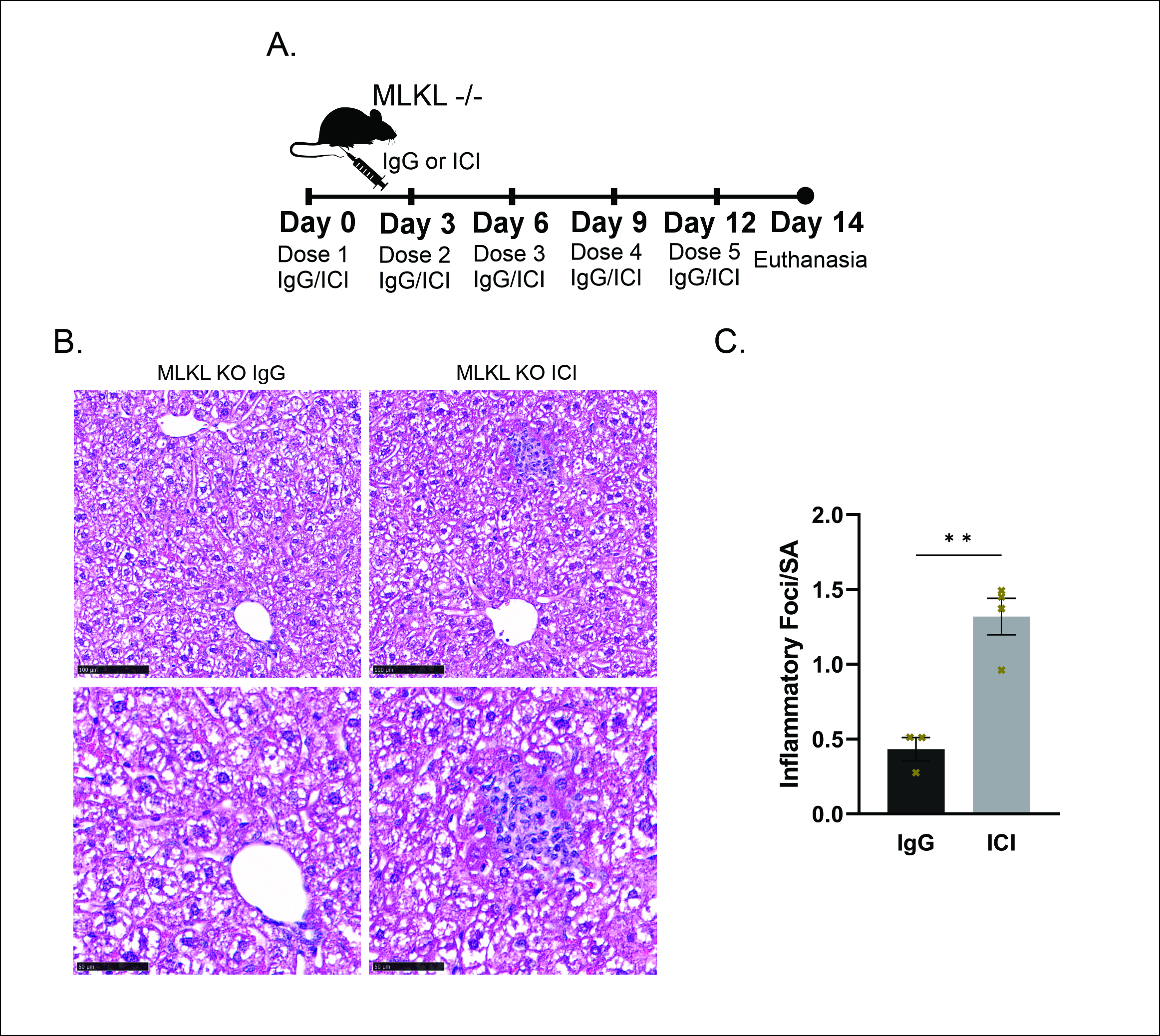

Supplement: Supplementary file 3 — Supplementary Figure 2 [file 41419_2024_6535_MOESM3_ESM.tif]

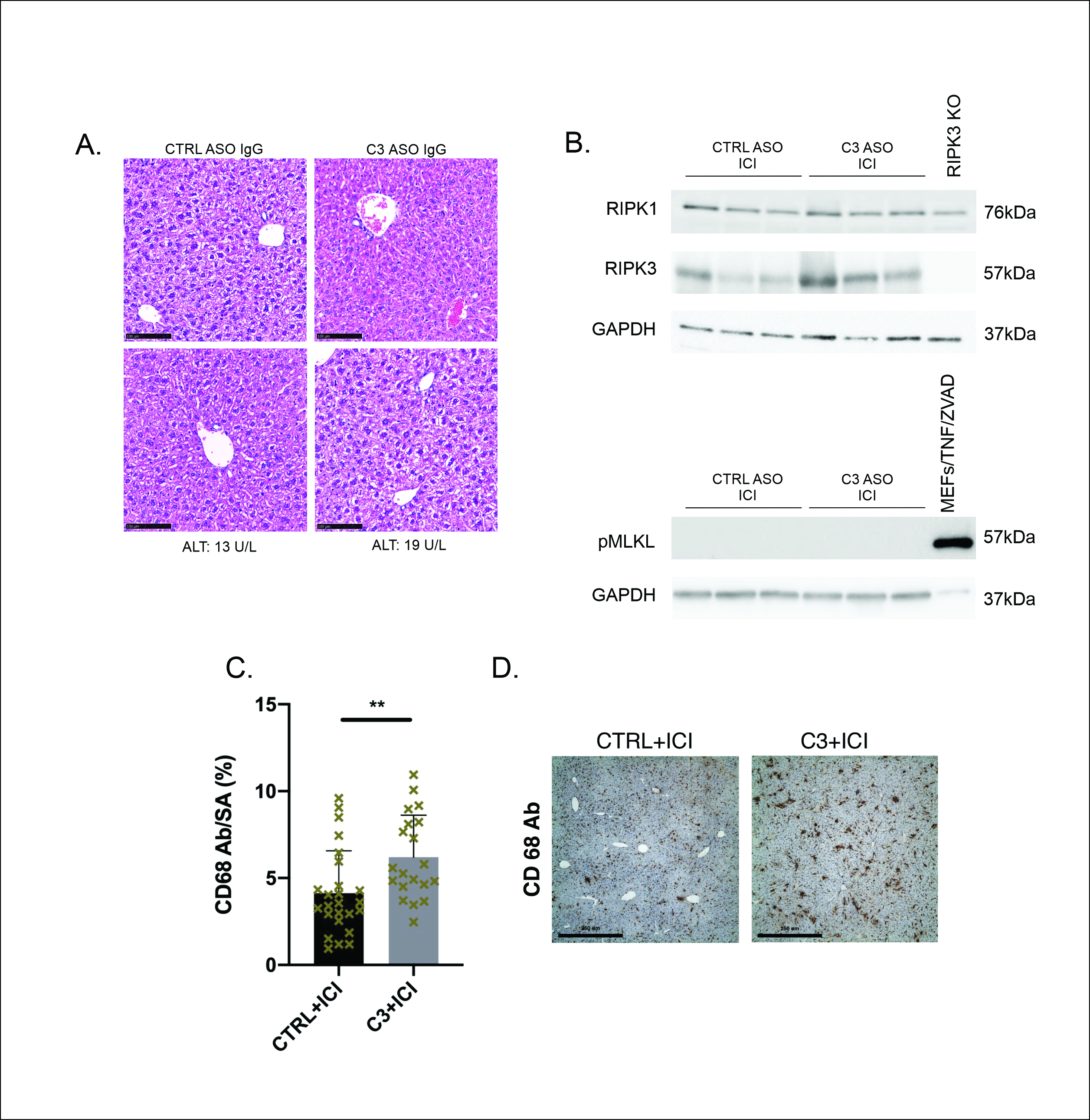

Supplement: Supplementary file 4 — Supplementary Figure 3 [file 41419_2024_6535_MOESM4_ESM.tif]

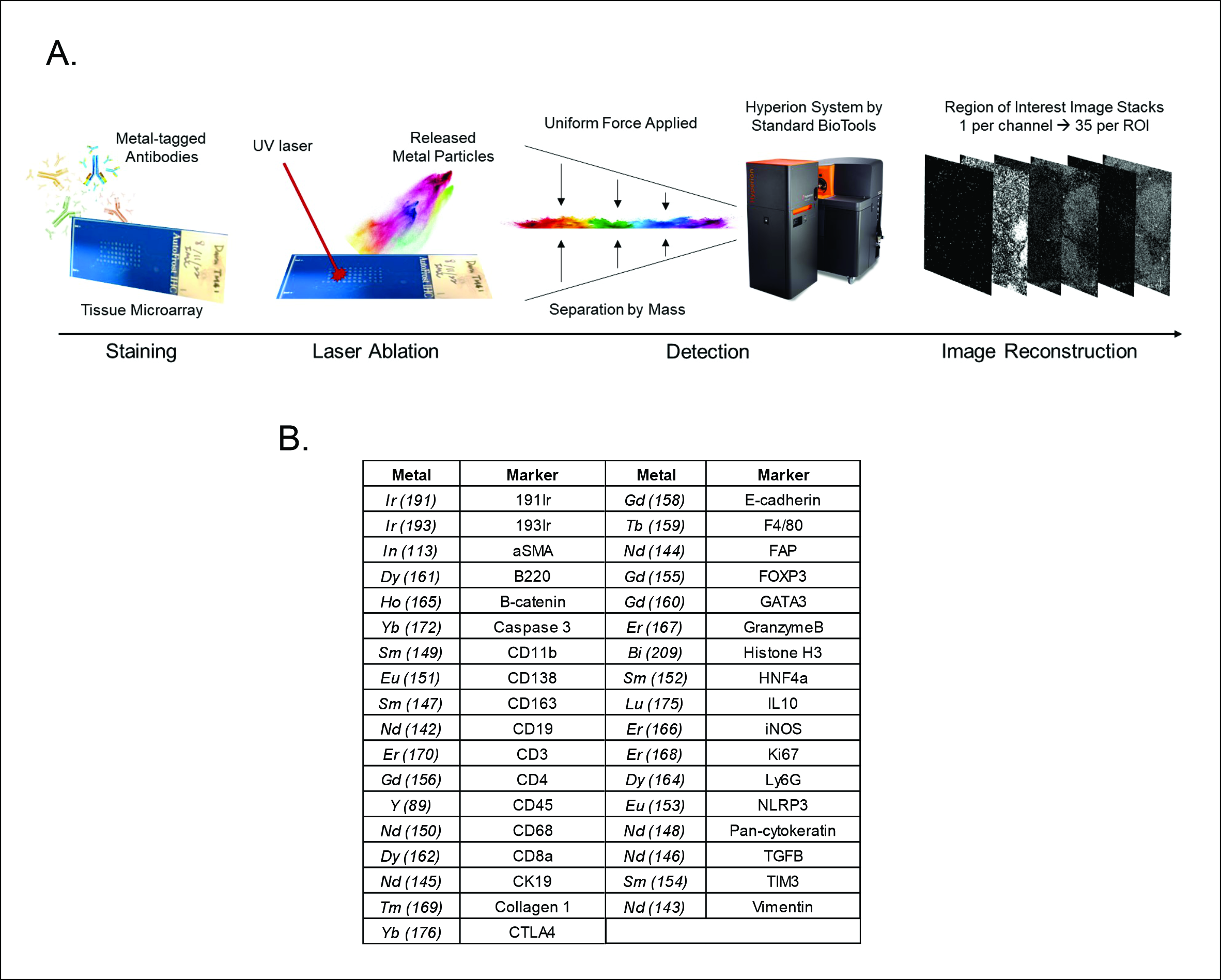

Supplement: Supplementary file 5 — Supplementary Figure 4 [file 41419_2024_6535_MOESM5_ESM.tif]
