# Supplementary material for: Innate and adaptive immune cell interaction drives inflammasome activation and hepatocyte apoptosis in murine liver injury from immune checkpoint inhibitors
Source: Cell Death Dis. 2024 Feb 14;15(2):140. doi: 10.1038/s41419-024-06535-7 (PMC10866933; doi:10.1038/s41419-024-06535-7)
Supplement: Supplementary file 6 — Authorship change approval [file 41419_2024_6535_MOESM6_ESM.pdf]

FW: CDDIS-23-2227RR Initial Quality Check

From: Lily Dara dara@usc.edu
To: Layla Shojaie ls_713@usc.edu
Date: Thu, Feb 1, 2024, 08:20

From: Lily Dara dara@usc.edu
To: Layla Shojaie ls_713@usc.edu, Bogdanov, Jacob Jacob.Bogdanov@med.usc.edu, Helia Alavifard alavifar@usc.edu, Mahmoud Galal Moawad Mohamed mm_623@usc.edu, Aria Baktash ab_261@usc.edu
Cc: myraali121@gmail.com , Mahov, Simeon Simeon.Mahov@cshs.org, Sue Murray SMurray@ionisph.com, Kanel, Gary Gary.Kanel@med.usc.edu, Zhang-Xu Liu zxliu@usc.edu, Ito, Fumito Fumito.Ito@med.usc.edu, In, Gino K Gino.In@med.usc.edu, Merchant, Akil Akil.Merchant@cshs.org, William Stohl stohl@usc.edu, Dara, Lily lily.dara@med.usc.edu
Date: Thu, Feb 1, 2024, 09:07
1.31.24 ILICI Final Merged file SUBMISSION_Resized.pdf 1.8 MB

Dear Co-authors,
We **revised the author list** when we resubmitted the CDD manuscript by adding new lab members and collaborators who contributed to the paper.
The journal requires that every one of you including newly added authors to **"REPLY ALL"** to this email and write the statement **" I approve the change in authorship list".**

See item 2. below
Thanks
Lily

From: Layla Shojaie ls_713@usc.edu
To: Lily Dara dara@usc.edu, Bogdanov, Jacob Jacob.Bogdanov@med.usc.edu, Helia Alavifard alavifar@usc.edu, Mahmoud Galal Moawad Mohamed mm_623@usc.edu, Aria Baktash ab_261@usc.edu
Cc: myraali121@gmail.com , Mahov, Simeon Simeon.Mahov@cshs.org, Sue Murray SMurray@ionisph.com, Kanel, Gary Gary.Kanel@med.usc.edu, Zhang-Xu Liu zxliu@usc.edu, Ito, Fumito Fumito.Ito@med.usc.edu, In, Gino K Gino.In@med.usc.edu,

Merchant, Akil Akil.Merchant@cshs.org, William Stohl stohl@usc.edu, Dara, Lily lily.dara@med.usc.edu
Date: Thu, Feb 1, 2024, 09:09

Hi,

I approve the change in authorship list.
Thanks,
Layla Shojaie

Layla Shojaie M.D.
Postdoctoral Fellow
Department of Medicine
Division of GI/Liver
Keck School of Medicine
University of Southern California
2011 Zonal Ave HMR 512
Los Angeles, CA 90033
(323) 442-1584
Layla.shojaie@usc.edu

From: myra ali myraali121@gmail.com
To: Lily Dara dara@usc.edu
Cc: Aria Baktash ab_261@usc.edu, Bogdanov, Jacob Jacob.Bogdanov@med.usc.edu, Dara, Lily lily.dara@med.usc.edu, Helia Alavifard alavifar@usc.edu, In, Gino K Gino.In@med.usc.edu, Ito, Fumito Fumito.Ito@med.usc.edu, Kanel, Gary Gary.Kanel@med.usc.edu, Layla Shojaie ls_713@usc.edu, Mahmoud Galal Moawad Mohamed mm_623@usc.edu, Mahov, Simeon Simeon.Mahov@cshs.org, Merchant, Akil Akil.Merchant@cshs.org, Sue Murray SMurray@ionisph.com, William Stohl stohl@usc.edu, Zhang-Xu Liu zxliu@usc.edu
Date: Thu, Feb 1, 2024, 09:11

Hello everyone,

I approve the change in authorship list.

Thank you everyone for letting me be a part of this project!!

Sincerely,

From: Ito, Fumito Fumito.Ito@med.usc.edu
To: Lily Dara dara@usc.edu, Layla Shojaie ls_713@usc.edu
Date: Thu, Feb 1, 2024, 09:13

I approve the change in authorship list.
Thanks.

Fumito Ito, MD, PhD, FACS
Associate Professor of Surgery
Leader of the Translational and Clinical Sciences Research (TACS) Program
Director of the Flow Cytometry and Immune Monitoring Core
Norris Comprehensive Cancer Center
University of Southern California
KECK Medicine of USC
1450 Biggy St. NRT 3505 Los Angeles, CA 90033
fumito.ito@med.usc.edu
TEL: (323) 929-9429 I Fax: (323) 865-0164

From: Aria Baktash ab_261@usc.edu
To: Layla Shojaie ls_713@usc.edu, Lily Dara lily.dara@usc.edu, Bogdanov, Jacob Jacob.Bogdanov@med.usc.edu, Helia Alavifard alavifar@usc.edu, Mahmoud Galal Moawad Mohamed mm_623@usc.edu
Cc: myraali121@gmail.com , Mahov, Simeon Simeon.Mahov@cshs.org, Sue Murray SMurray@ionisph.com, Kanel, Gary Gary.Kanel@med.usc.edu, Zhang-Xu Liu zxliu@usc.edu, Ito, Fumito Fumito.Ito@med.usc.edu, In, Gino K Gino.In@med.usc.edu, Merchant, Akil Akil.Merchant@cshs.org, William Stohl stohl@usc.edu, Dara, Lily lily.dara@med.usc.edu
Date: Thu, Feb 1, 2024, 09:23

Hi everyone,

I approve the change in authorship list.
Thanks,
Aria Baktash

From: Bogdanov, Jacob Jacob.Bogdanov@med.usc.edu
To: Aria Baktash ab_261@usc.edu
Cc: Layla Shojaie ls_713@usc.edu, Lily Dara lily.dara@usc.edu, Helia Alavifard alavifar@usc.edu, Mahmoud Galal Moawad Mohamed mm_623@usc.edu, myraali121@gmail.com , Mahov, Simeon Simeon.Mahov@cshs.org, Sue Murray SMurray@ionisph.com, Kanel, Gary Gary.Kanel@med.usc.edu, Zhang-Xu Liu zxliu@usc.edu, Ito, Fumito Fumito.Ito@med.usc.edu, In, Gino K Gino.In@med.usc.edu, Merchant, Akil M.D. Akil.Merchant@cshs.org, William Stohl stohl@usc.edu, Dara, Lily Lily.Dara@med.usc.edu
Date: Thu, Feb 1, 2024, 09:25

I approve the change in authorship list.

Thank you,
Jacob Bogdanov

On Feb 1, 2024, at 9:23 AM, Aria Baktash <ab_261@usc.edu> wrote:

Hi everyone,

I approve the change in authorship list.
Thanks,
Aria Baktash

From: William Stohl stohl@usc.edu
To: Bogdanov, Jacob Jacob.Bogdanov@med.usc.edu, Aria Baktash ab_261@usc.edu
Cc: Layla Shojaie ls_713@usc.edu, Lily Dara lily.dara@usc.edu, Helia Alavifard alavifar@usc.edu, Mahmoud Galal Moawad Mohamed mm_623@usc.edu, myraali121@gmail.com , Mahov, Simeon Simeon.Mahov@cshs.org, Sue Murray SMurray@ionisph.com, Kanel, Gary Gary.Kanel@med.usc.edu, Zhang-Xu Liu zxliu@usc.edu, Ito, Fumito Fumito.Ito@med.usc.edu, In, Gino K Gino.In@med.usc.edu, Merchant, Akil M.D. Akil.Merchant@cshs.org, Dara, Lily Lily.Dara@med.usc.edu
Date: Thu, Feb 1, 2024, 09:27

I approve the change in authorship list.

WS

William Stohl, MD, PhD

Master, American College of Rheumatology

Professor of Medicine

Chief, Division of Rheumatology

University of Southern California Keck School of Medicine

2011 Zonal Ave. HMR 711

Los Angeles, CA 90033

323-442-1946 (office)

323-442-2874 (FAX)

From: Zhang-Xu Liu zxliu@usc.edu
To: Lily Dara dara@usc.edu, Layla Shojaie ls_713@usc.edu, Bogdanov, Jacob Jacob.Bogdanov@med.usc.edu, Helia Alavifard alavifar@usc.edu, Mahmoud Galal Moawad Mohamed mm_623@usc.edu, Aria Baktash ab_261@usc.edu
Cc: myraali121@gmail.com , Mahov, Simeon Simeon.Mahov@cshs.org, Sue Murray SMurray@ionisph.com, Kanel, Gary Gary.Kanel@med.usc.edu, Ito, Fumito Fumito.Ito@med.usc.edu, In, Gino K Gino.In@med.usc.edu, Merchant, Akil Akil.Merchant@cshs.org, William Stohl stohl@usc.edu, Dara, Lily lily.dara@med.usc.edu
Date: Thu, Feb 1, 2024, 09:29

I approve the change of author list.
Zhang-Xu Liu

Get [Outlook for iOS]

From: Merchant, Akil M.D. Akil.Merchant@cshs.org
To: Zhang-Xu Liu zxliu@usc.edu
Cc: Lily Dara dara@usc.edu, Layla Shojaie ls_713@usc.edu, Bogdanov, Jacob Jacob.Bogdanov@med.usc.edu, Helia Alavifard alavifar@usc.edu, Mahmoud Galal Moawad Mohamed mm_623@usc.edu, Aria Baktash ab_261@usc.edu, myraali121@gmail.com , Mahov, Simeon Simeon.Mahov@cshs.org, Sue Murray

SMurray@ionisph.com, Kanel, Gary gary.kanel@med.usc.edu, Ito, Fumito Fumito.Ito@med.usc.edu, In, Gino K gino.in@med.usc.edu, William Stohl stohl@usc.edu, Dara, Lily lily.dara@med.usc.edu
Date: Thu, Feb 1, 2024, 09:34

I approve revised authorship
Akil

On Feb 1, 2024, at 10:59 PM, Zhang-Xu Liu <zxliu@usc.edu> wrote:

CAUTION: External Sender
Do not click on links or open attachments unless you know the content is safe. Protect your username and password.

I approve the change of author list.
Zhang-Xu Liu

Get Outlook for iOS

From: Sue Murray SMurray@ionisph.com
To: Lily Dara dara@usc.edu, Layla Shojaie ls_713@usc.edu, Bogdanov, Jacob Jacob.Bogdanov@med.usc.edu, Helia Alavifard alavifar@usc.edu, Mahmoud Galal Moawad Mohamed mm_623@usc.edu, Aria Baktash ab_261@usc.edu
Cc: myraali121@gmail.com , Mahov, Simeon Simeon.Mahov@cshs.org, Kanel, Gary Gary.Kanel@med.usc.edu, Zhang-Xu Liu zxliu@usc.edu, Ito, Fumito Fumito.Ito@med.usc.edu, In, Gino K Gino.In@med.usc.edu, Merchant, Akil Akil.Merchant@cshs.org, William Stohl stohl@usc.edu, Dara, Lily lily.dara@med.usc.edu
Date: Thu, Feb 1, 2024, 09:58

Hi All,

I approve the change in authorship list.

Sue

Sue Murray
Director, Antisense Drug Discovery
smurray@ionisph.com
D: (760)603-2675 l M: (858)395-8113
2855 Gazelle Court, Carlsbad, CA 92010
ionispharma.com

From: Helia Alavifard alavifar@usc.edu
To: Sue Murray SMurray@ionisph.com, Lily Dara dara@usc.edu, Layla Shojaie ls_713@usc.edu, Bogdanov, Jacob Jacob.Bogdanov@med.usc.edu, Mahmoud Galal Moawad Mohamed mm_623@usc.edu, Aria Baktash ab_261@usc.edu
Cc: myraali121@gmail.com , Mahov, Simeon Simeon.Mahov@cshs.org, Kanel, Gary Gary.Kanel@med.usc.edu, Zhang-Xu Liu zxliu@usc.edu, Ito, Fumito Fumito.Ito@med.usc.edu, In, Gino K Gino.In@med.usc.edu, Merchant, Akil Akil.Merchant@cshs.org, William Stohl stohl@usc.edu, Dara, Lily lily.dara@med.usc.edu
Date: Thu, Feb 1, 2024, 11:34

Hi,

I approve the change in authorship list.

Thank you,
Helia Alavifard

Get Outlook for iOS

From: Mahov, Simeon Simeon.Mahov@cshs.org
To: Lily Dara dara@usc.edu, Layla Shojaie ls_713@usc.edu, Bogdanov, Jacob Jacob.Bogdanov@med.usc.edu, Helia Alavifard alavifar@usc.edu, Mahmoud Galal Moawad Mohamed mm_623@usc.edu, Aria Baktash ab_261@usc.edu
Cc: myraali121@gmail.com , Sue Murray SMurray@ionisph.com, Kanel, Gary Gary.Kanel@med.usc.edu, Zhang-Xu Liu zxliu@usc.edu, Ito, Fumito Fumito.Ito@med.usc.edu, In, Gino K gino.in@med.usc.edu, Merchant, Akil M.D. Akil.Merchant@cshs.org, William Stohl stohl@usc.edu, Dara, Lily

lily.dara@med.usc.edu
Date: Thu, Feb 1, 2024, 11:47

I approve the change in authorship list

From: In, Gino K Gino.In@med.usc.edu
To: Mahov, Simeon Simeon.Mahov@cshs.org, Lily Dara dara@usc.edu, Layla Shojaie ls_713@usc.edu, Bogdanov, Jacob Jacob.Bogdanov@med.usc.edu, Helia Alavifard alavifar@usc.edu, Mahmoud Galal Moawad Mohamed mm_623@usc.edu, Aria Baktash ab_261@usc.edu
Cc: myraali121@gmail.com , Sue Murray SMurray@ionisph.com, Kanel, Gary Gary.Kanel@med.usc.edu, Zhang-Xu Liu zxliu@usc.edu, Ito, Fumito Fumito.Ito@med.usc.edu, Merchant, Akil M.D. Akil.Merchant@cshs.org, William Stohl stohl@usc.edu, Dara, Lily lily.dara@med.usc.edu
Date: Thu, Feb 1, 2024, 12:05

I approve the change in authorship list

From: Mahmoud Galal Moawad Mohamed mm_623@usc.edu
To: Layla Shojaie ls_713@usc.edu
Date: Thu, Feb 1, 2024, 12:41

Mahmoud Mohamed, M.D.
Postdoctoral Fellow
Department of Medicine, Division of GI/Liver
Keck School of Medicine
University of Southern California
2011 Zonal Ave HMR 512
Los Angeles, CA 90033
(714) 726-0733
Mahmoudgalalmoawad.Mohamed@usc.edu

From: Kanel, Gary Gary.Kanel@med.usc.edu
To: Lily Dara dara@usc.edu, Layla Shojaie ls_713@usc.edu, Bogdanov, Jacob Jacob.Bogdanov@med.usc.edu, Helia Alavifard alavifar@usc.edu, Mahmoud Galal Moawad Mohamed mm_623@usc.edu, Aria Baktash ab_261@usc.edu

Cc: myraali121@gmail.com , Mahov, Simeon Simeon.Mahov@cshs.org, Sue Murray SMurray@ionisph.com, Zhang-Xu Liu zxliu@usc.edu, Ito, Fumito Fumito.Ito@med.usc.edu, In, Gino K Gino.In@med.usc.edu, Merchant, Akil M.D. Akil.Merchant@cshs.org, William Stohl stohl@usc.edu, Dara, Lily Lily.Dara@med.usc.edu
Date: Thu, Feb 1, 2024, 12:55

I approve the change in authorship list

Thank you

Gary Kanel

From: Lily Dara dara@usc.edu
To: Mahmoud Galal Moawad Mohamed mm_623@usc.edu, Helia Alavifard alavifar@usc.edu, Aria Baktash ab_261@usc.edu, Layla Shojaie ls_713@usc.edu
Cc: myraali121@gmail.com , Mahov, Simeon Simeon.Mahov@cshs.org, Sue Murray SMurray@ionisph.com, Kanel, Gary Gary.Kanel@med.usc.edu, Zhang-Xu Liu zxliu@usc.edu, Ito, Fumito Fumito.Ito@med.usc.edu, In, Gino K Gino.In@med.usc.edu, Merchant, Akil Akil.Merchant@cshs.org, William Stohl stohl@usc.edu, Dara, Lily lily.dara@med.usc.edu
Date: Thu, Feb 1, 2024, 14:48

I approve the change in authorship list

From: Lily Dara dara@usc.edu
To: Layla Shojaie ls_713@usc.edu
Date: Thu, Feb 1, 2024, 14:49

---

18 Emails
