# Supplementary material for: Innate and adaptive immune cell interaction drives inflammasome activation and hepatocyte apoptosis in murine liver injury from immune checkpoint inhibitors
Source: Cell Death Dis. 2024 Feb 14;15(2):140. doi: 10.1038/s41419-024-06535-7 (PMC10866933; doi:10.1038/s41419-024-06535-7)

Original Western Blots
Supplementary Material

Figure 2A

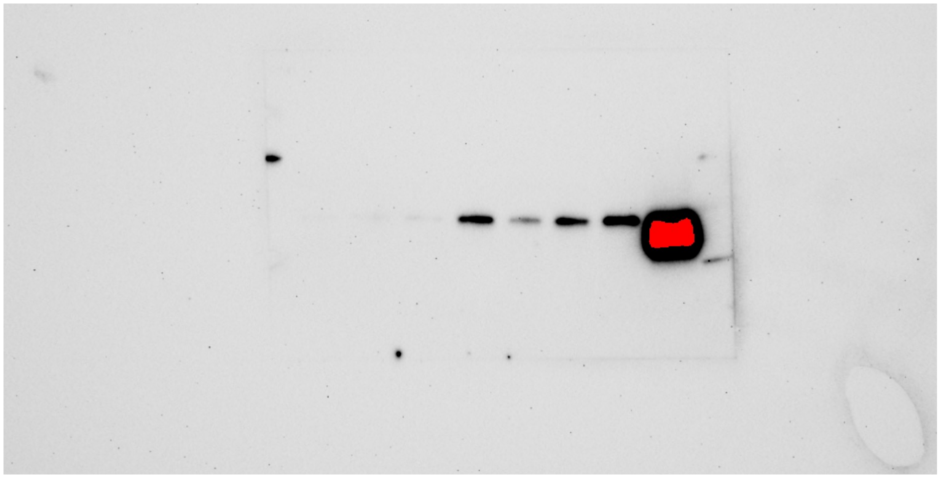

Cleaved caspase-3

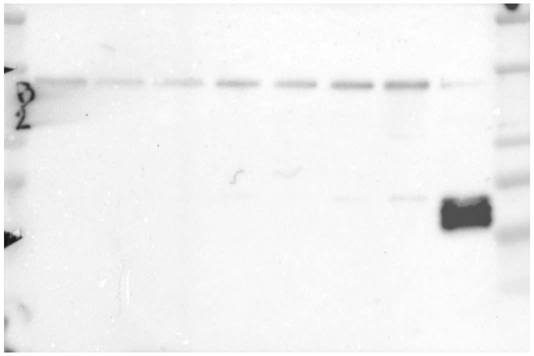

GAPDH

Figure 2D - top

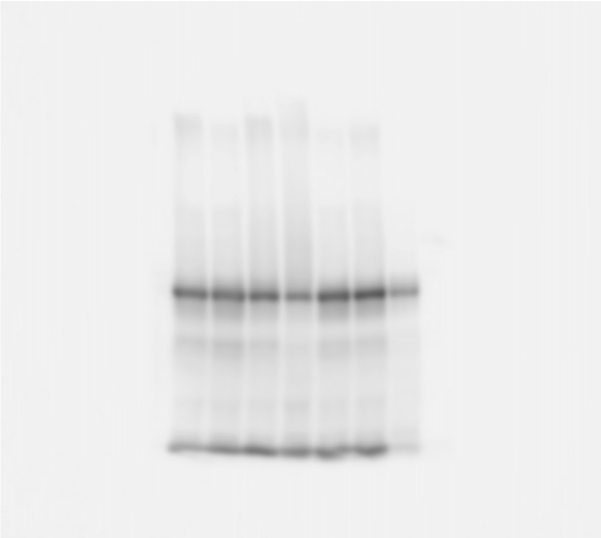

RIPK1

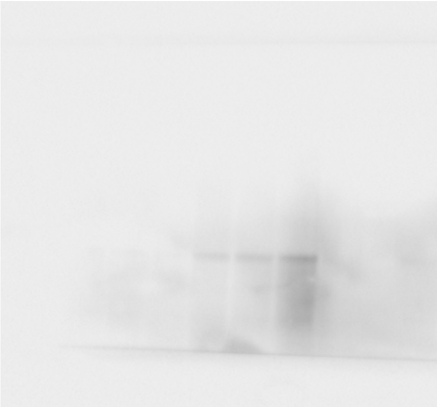

RIPK3

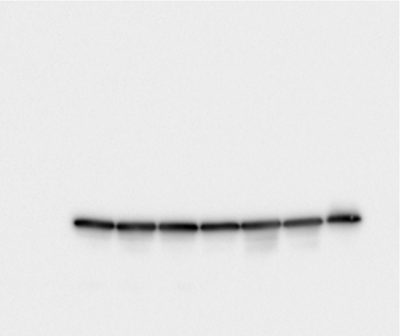

GAPDH

Figure 2D - bottom

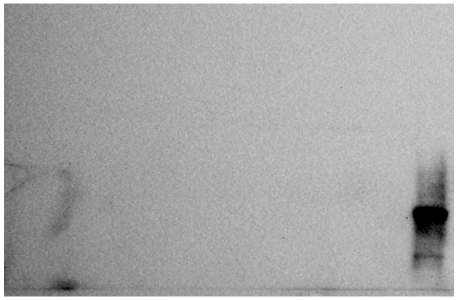

pMLKL

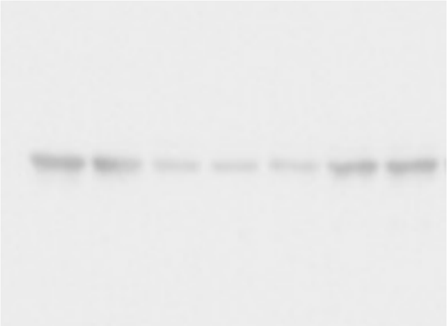

GAPDH

Figure 3A

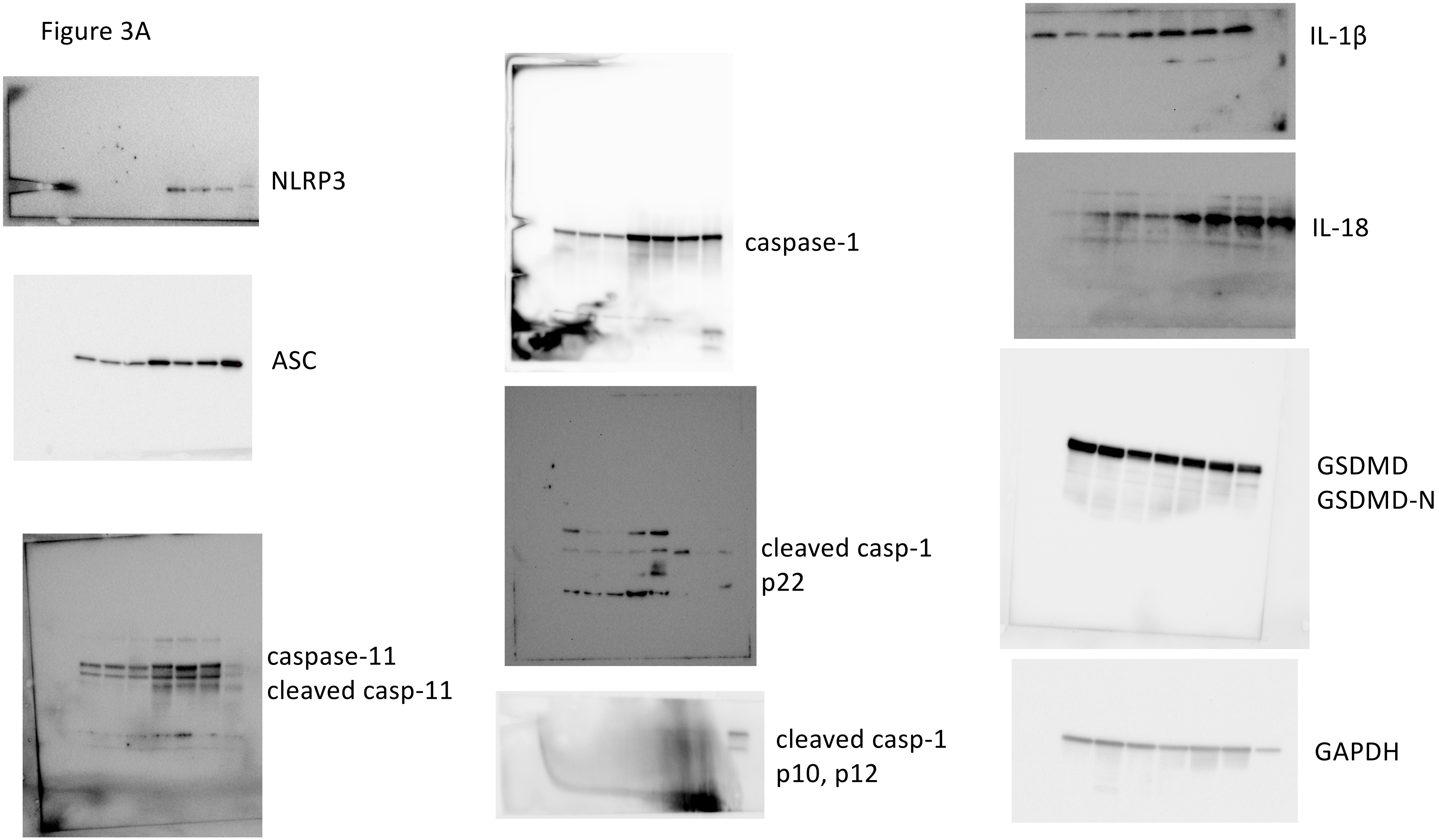
NLRP3
ASC
caspase-11
cleaved casp-11
caspase-1
cleaved casp-1
p22
cleaved casp-1
p10, p12
IL-1β
IL-18
GSDMD
GSDMD-N
GAPDH

Figure 4A

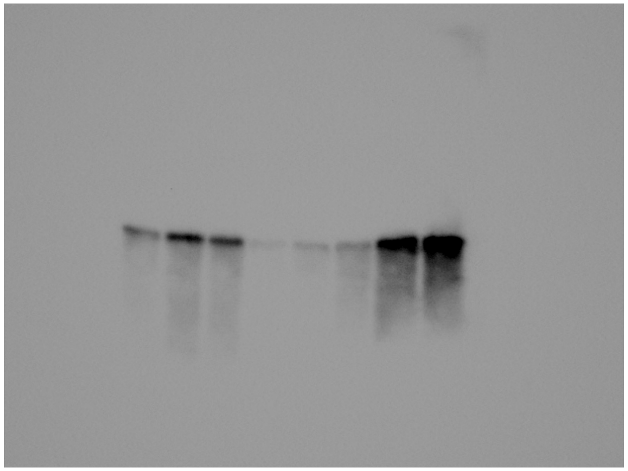

caspase-3

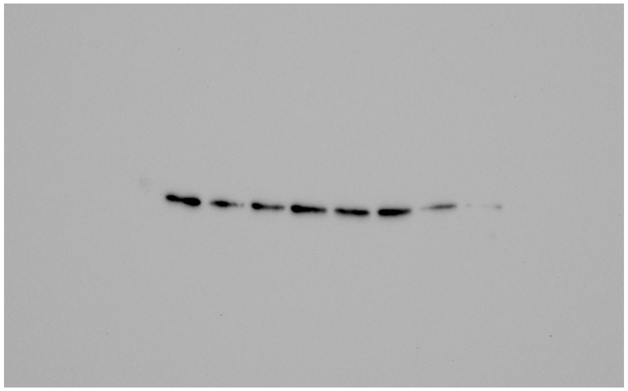

GAPDH

Figure 5A

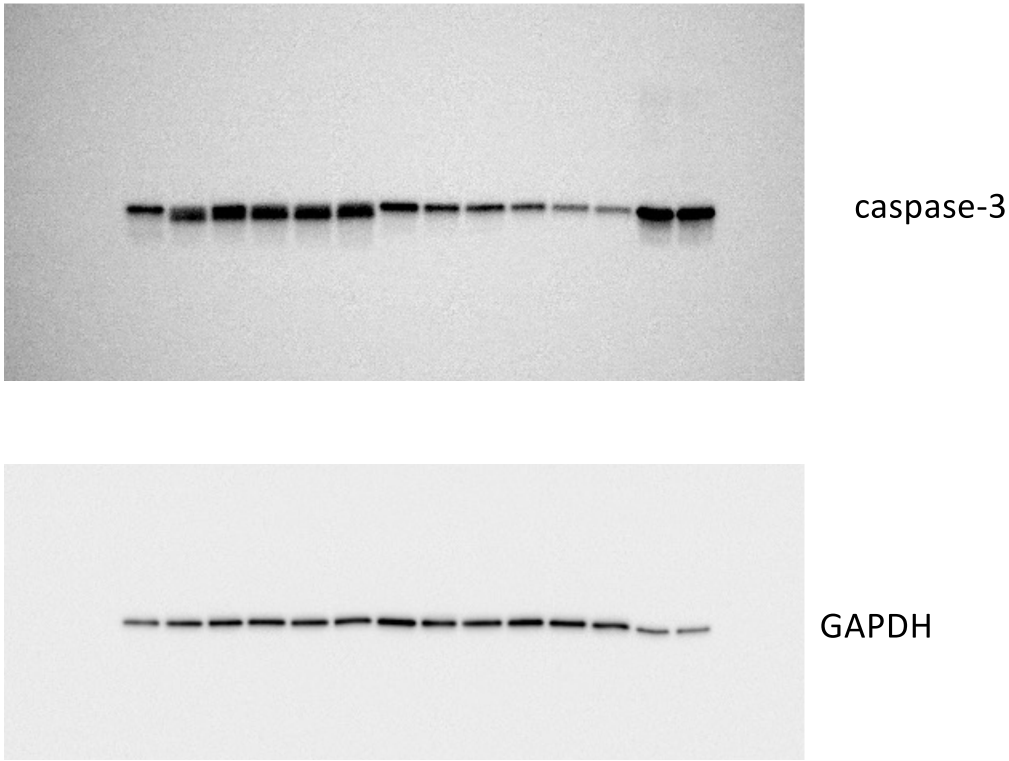

Figure 5G

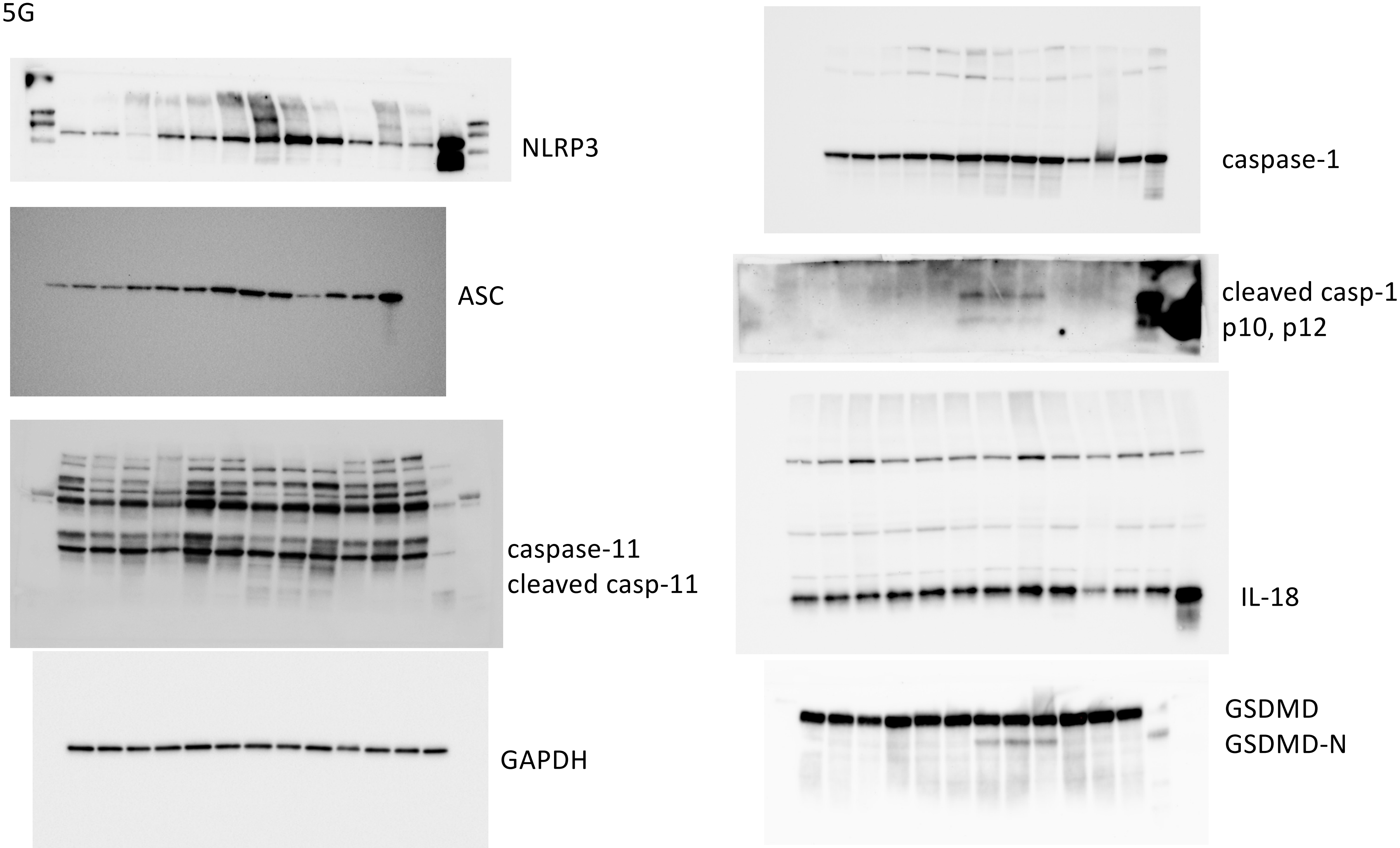
NLRP3
ASC
caspase-11
cleaved casp-11
GAPDH
caspase-1
cleaved casp-1
p10, p12
IL-18
GSDMD
GSDMD-N

Supplemental Figure 3B - top

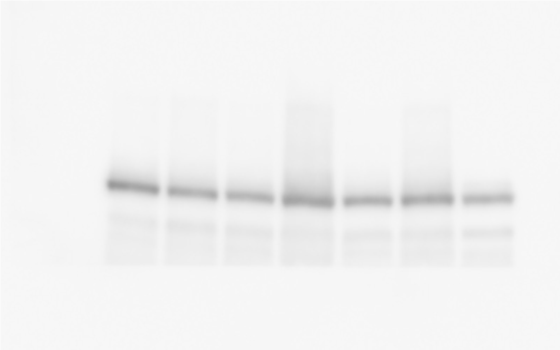

RIPK1

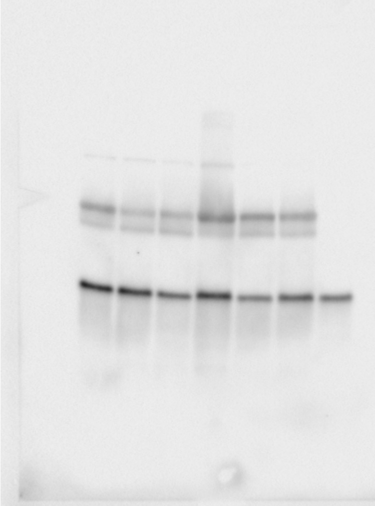

RIPK3

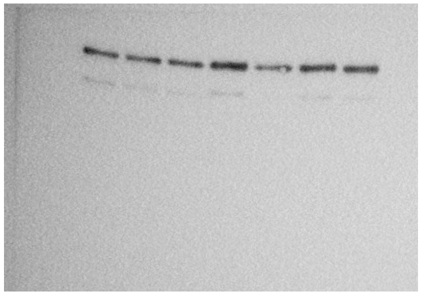

GAPDH

Supplemental Figure 3B - bottom

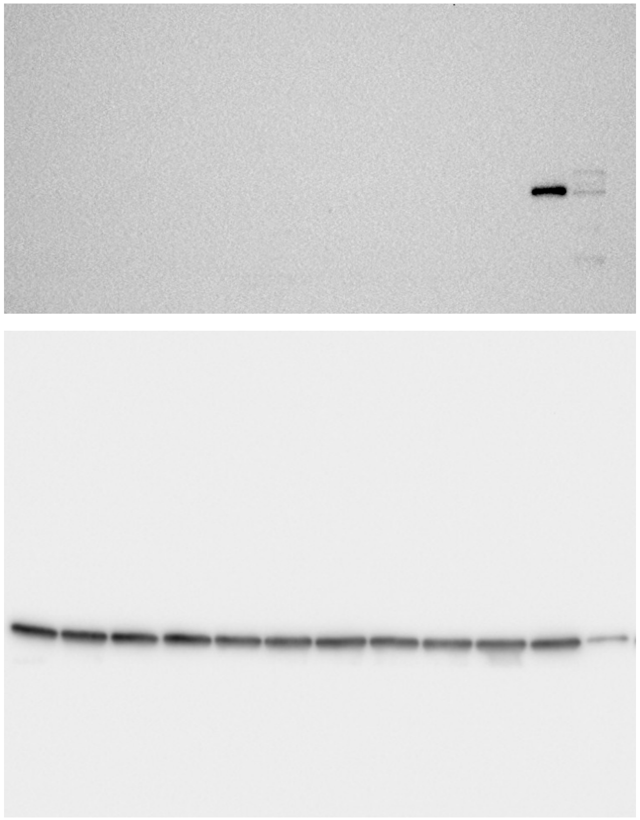

Supplement: Supplementary file 7 — Original full-length Western Blots [file 41419_2024_6535_MOESM7_ESM.pdf]
